# Proteomic Analysis Uncovers Enhanced Inflammatory Phenotype and Distinct Metabolic Changes in IDH1 Mutant Glioma Cells

**DOI:** 10.3390/ijms26189075

**Published:** 2025-09-18

**Authors:** Sigrid Ravn Berg, Alessandro Brambilla, Lars Hagen, Animesh Sharma, Cathrine Broberg Vågbø, Nina Beate Liabakk, Miroslava Kissova, Miquel Arano Barenys, Magnar Bjørås, Sverre Helge Torp, Geir Slupphaug

**Affiliations:** 1 Department of Clinical and Molecular Medicine, Norwegian University of Science and Technology, 7491 Trondheim, Norway; sigrbe@ntnu.no (S.R.B.); lars.hagen@ntnu.no (L.H.); animesh.sharma@ntnu.no (A.S.); cathrine.b.vagbo@ntnu.no (C.B.V.); nina.beate.liabakk@ntnu.no (N.B.L.); miroslava.kissova@ntnu.no (M.K.); miquela@stud.ntnu.no (M.A.B.); magnar.bjoras@ntnu.no (M.B.); sverre.torp@ntnu.no (S.H.T.); 2 Clinic of Laboratory Medicine, St. Olavs Hospital, 7491 Trondheim, Norway; 3 The Proteomics and Modomics Core Facility, PROMEC, at NTNU and the Central Norway Regional Health Authority, 7491 Trondheim, Norway; 4 Department of Microbiology, Oslo University Hospital and University of Oslo, 0372 Oslo, Norway; 5 Norway Centre of Embryology (CRESCO), University of Oslo, 0313 Oslo, Norway; 6 Department of Pathology, St. Olavs Hospital, Trondheim University Hospital, 7006 Trondheim, Norway

**Keywords:** glioma biomarkers, isocitrate dehydrogenase mutations, proteomics, antigen presentation, interferon signaling, DNA repair, RNA, DNA and histone demethylases

## Abstract

Isocitrate dehydrogenase 1 (IDH1) mutations are key drivers of glioma biology, influencing tumor aggressiveness and treatment response. To elucidate their molecular impact, we performed proteome analysis on patient-derived (PD) and U87MG glioma cell models with either mutant or wild-type IDH1. We quantified over 6000 protein groups per model, identifying 1594 differentially expressed proteins in PD-AS (IDH1_MUT_) vs. PD-GB (IDH1_WT_) and 904 in U87_MUT_ vs. U87_WT_. Both IDH1_MUT_ models exhibited enhanced MHC antigen presentation and interferon signaling, indicative of an altered immune microenvironment. However, metabolic alterations were model-dependent: PD-AS cells shifted toward glycolysis and purine salvage, while U87_MUT_ cells retained oxidative phosphorylation, potentially due to D2-hydroxyglutarate (2OHG)-mediated HIF1A stabilization. We also observed a predominance of downregulated DNA repair proteins in IDH1_MUT_ models, particularly those involved in homologous recombination. In contrast, RB1 and ASMTL were strongly upregulated in both IDH1_MUT_ models, implicating them in DNA repair and cellular stress responses. We also found distinct expression patterns of proteins regulating histone methylation in IDH1_MUT_ cells, favoring increased methylation of H3K4, H3K9, and H3K36. A key driver of this may be the upregulation of SETD2 in PD-AS, an H3K4 and H3K36 trimethyltransferase linked to the recruitment of HIF1A as well as DNA mismatch repair proteins. This study uncovers candidate biomarkers and pathways relevant to glioma progression and therapeutic targeting, but also underscores the complexity of predicting glioma pathogenesis and treatment responses based on IDH1 mutation status. While proteome profiling provides valuable insights, a comprehensive understanding of IDH1_MUT_ gliomas will likely require integrative multi-omics approaches, including DNA/RNA methylation profiling, histone and protein post-translational modification analyses, and targeted DNA damage and repair assays.

## 1. Introduction

Glioblastoma (GB) is the most prevalent primary malignant brain tumor in adults, accounting for approximately 50% of all gliomas [1]. According to the 2021 WHO classification, tumors formerly known as adult-type diffuse gliomas are now categorized into three types: astrocytoma, isocitrate dehydrogenase (IDH) mutant; oligodendroglioma, IDH mutant and 1p/19q codeleted; and glioblastoma, IDH wild-type [2]. IDH-mutant astrocytomas usually receive radiotherapy in conjunction with adjuvant temozolomide (TMZ), whereas glioblastomas receive radiotherapy and either concurrent or adjuvant TMZ [3]. Despite these treatments, glioblastomas have a median survival of only 12–15 months. Although IDH-mutant gliomas, often classified as low-grade gliomas (LGGs), are associated with a better prognosis, the majority ultimately recur. IDH missense mutations primarily affect arginines in the isocitrate-binding pocket. Most commonly, R132 in cytosolic IDH1 or the analogous R172 in mitochondrial IDH2 are affected. Change of either arginine to a less polar residue, like histidine, glycine, cysteine, or lysine, reduces its affinity for isocitrate while increasing its preference for α-ketoglutarate (2OG) and NADPH [4]. Wild-type IDH1 exists in the cytoplasm as homodimers that produce 2OG by using NADP+ as a cofactor. Since IDH1 mutations are monoallelic, this results in a mix of mutant and wild-type dimers. Such dimers irreversibly convert 2OG to the D2-enantiomer of hydroxyglutarate (2OHG in the following) and use NADPH as cofactor. In these tumor cells, 2OHG can rise to millimolar concentrations, while 2OG is strongly reduced. 2OHG competitively inhibits 2OG-dependent enzymes such as histone demethylases and TET- and ALKBH-family enzymes, resulting in RNA, DNA, and histone hypermethylation, which are epigenetic regulatory mechanisms associated with tumorigenesis. 2OHG is thus classified as an oncometabolite (5). Interestingly, astrocytomas harboring the IDH1 R132H mutation (~90%) display lower genomic methylation compared to non-R132H mutations, which signifies a poorer prognosis [5].

While numerous studies have sought to distinguish IDH-mutant from IDH-wild-type tumors using genomic and transcriptomic analyses, these approaches may not fully capture the alterations occurring at the proteomic level. This discrepancy is partly due to the well-documented poor correlation between mRNA and protein expression [6], which is particularly relevant for brain tumors [7,8,9]. To this end, we have subjected patient-derived tumor cells from an R132C IDH1 mutant astrocytoma (grade III) patient (PD-AS) and an IDH1 wild-type (grade IV) glioblastoma patient (PD-GB) to label-free quantitative proteome profiling. Furthermore, to identify potentially differentially expressed proteins (DEPs) directly associated with mutant IDH1, we have compared the DEPs from the patient cell lines with those found in isogenic wild-type U87MG cells (U87_WT_) compared to U87MG cells expressing monoallelic IDH1 R132H (U87_MUT_). Among the biological processes most similarly affected by the IDH1 mutation in our cell models was upregulated interferon signaling, and antigen processing and presentation via MHC complexes, implying that the IDH1 mutation status may be directly relevant for therapeutic strategies involving immunotherapy [10,11].

## 2. Results and Discussion

### 2.1. Common DEPs in IDH1 Mutant Versus IDH1 Wild-Type Glioma Cells Are Associated with Tumor Aggressiveness and Response to Treatment

Proteins extracted from PD-GB, PD-AS, U87_WT_, and U87_MUT_ cells were subjected to label-free quantitative (LFQ) LC-MS/MS proteome analysis. In total, we quantified 6334 and 6359 protein groups in PD-AS and PD-GB, respectively, of which 6148 protein groups were common to both (Supplementary Table S1). In U87_MUT_ and U87_WT_, we quantified 5793 and 5789 protein groups, respectively, of which 5699 protein groups were common to both (Supplementary Table S2).

**Figure 1:**
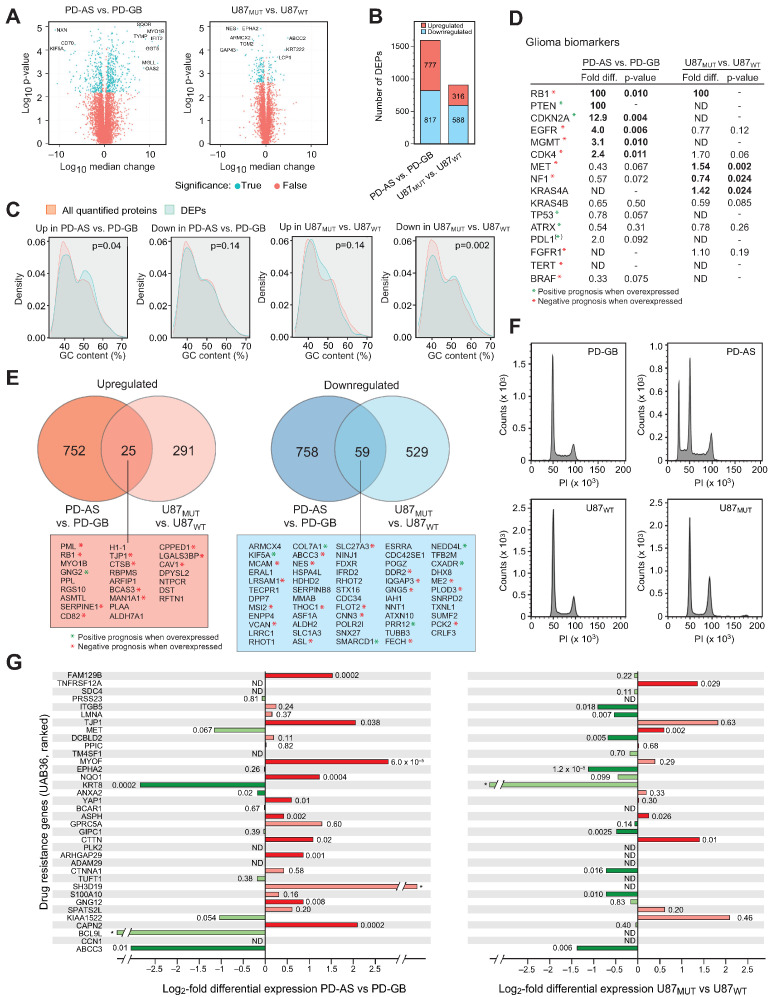
Differentially expressed proteins in the IDH1 mutant PD-AS and U87_MUT_ cells compared to their IDH1 wild-type counterparts. (**A**) Volcano plots of DEPs in PD-AS vs. PD-GB (left panel) and U87_MUT_ vs. U87_WT_ (right panel). (**B**) The number of up- and downregulated DEPs in PD-AS vs. PD-GB and in U87_MUT_ vs. U87_WT_. (**C**) ShinyGO analysis of the GC-content of the genes encoding DEPs compared to the genes encoding all quantified proteins in the dataset. (**D**) Expression of proteins commonly used as prognostic and/or therapeutic biomarkers in glioma. ND: Not detected. Green stars represent positive prognosis when overexpressed, red stars represent negative prognosis when overexpressed. (**E**) Venn diagrams illustrating common up- and downregulated DEPs in the two model systems. Common DEPs are listed in the bottom boxes, ranging from most upregulated (red box) to most downregulated (blue box). Green stars represent positive prognosis when overexpressed, red stars represent negative prognosis when overexpressed. (**F**) Cell cycle analysis of freely cycling PD-GB and PD-AS cells (upper panels) and U87_WT_ and U87_MUT_ cells (bottom panels). (**G**) The majority of the DEPs encoded by the drug resistance genes in the UAB36 panel were upregulated in the IDH1 mutant PD-AS cells compared to IDH wild-type PD-GB, whereas most of the UAB36 genes were downregulated in U87_MUT_ vs. U87_WT_. *; detected only in one of the cell pairs, thus reported as non-significant.

Among the most strongly upregulated proteins in PD-AS compared to PD-GB were SQOR, MYO1B, TYMP, IFIT2, GGT5, MGLL, and OAS2 (Figure 1A, left panel). Several of these have documented relevance in glioma biology. MYO1B has been shown to promote glioblastoma angiogenesis [12], while TYMP (thymidine phosphorylase) supports glioma stem cell proliferation and self-renewal [13]. The >100-fold higher expression of interferon-induced IFIT2 in PD-AS relative to PD-GD may be linked to the presence of PTEN, which was only detected in PD-AS (Supplementary Table S1). IFIT2 expression has been correlated with PTEN status at the transcript level, and PTEN loss was associated with reduced glioma immunogenicity, including decreased CD8 infiltration and lower interferon-inducible gene expression [14]. Consistently, OAS2, another interferon-stimulated gene, was also among the most upregulated in PD-AS (Figure 1A). Conversely, CD70 was ~90-fold lower in PD-AS compared to PD-GB. Given that CD70 promotes immune suppression by inducing CD8+ T-cell death and its expression correlates with poor survival in glioma [15], this downregulation may reflect a more immunologically permissive phenotype in PD-AS.

The U87WT vs. U87MUT comparison revealed fewer outlier proteins overall (Figure 1A, right panel), consistent with the isogenic nature of this model. Notably, ABCC2, a transporter linked to multidrug resistance, was upregulated ~28-fold in U87_MUT_, and LCP1 (plastin-2) was increased ~6-fold. LCP1 knockdown has been reported to reduce glioma cell viability and invasiveness [16]. In contrast, the intermediate filament protein nestin (NES), a widely used glioma stem cell marker associated with increased malignancy and invasiveness [17], was ~78-fold lower in U87_MUT_ compared to U87_WT_. Interestingly, a recent IHC-based study found NES expressions to correlate positively with IDH1 R132H mutation status in gliomas [18]. The molecular mechanisms underlying this apparently contrasting finding remain to be further investigated. The divergence between our proteomic results and these tissue-level findings suggests that the regulation of NES in IDH1-mutant gliomas may be context-dependent, influenced by model system and microenvironment, and merits further investigation.

To identify proteins and pathways potentially involved in tumor aggressiveness and treatment responses, we selected differentially expressed proteins (DEPs) for further analysis using a statistical threshold of *p* < 0.05 and an absolute log_2_ LFQ difference > |0.585|, corresponding to a 1.5-fold change in expression (up- or downregulated). In addition, proteins that were consistently quantified in only one of the cell lines but not the other were also classified as DEPs. These proteins were assigned differential expression ratios of either 0.01 or 100, as presented in Supplementary Tables S1 and S2. Although statistical significance could not be calculated for these proteins, they may still represent biologically relevant processes in the cells.

The filtering identified 1594 DEPs in PD-AS vs. PD-GB (777 upregulated, 817 downregulated) and 904 DEPS in U87_MUT_ vs. U87_WT_ (316 upregulated, 588 downregulated) (Figure 1B). The greater number of DEPs in the patient-derived comparison likely reflects differences in genetic background: the U87 system is isogenic, where variation is attributable mainly to the introduced IDH1 mutation, whereas the PD models originate from distinct patients with broader genomic divergence. This background also influences the directionality of changes. In U87_MUT_, downregulated DEPs were strongly overrepresented, consistent with the well-established DNA hypermethylation phenotype of IDH1 mutations being more apparent in an otherwise identical background. This was corroborated by ShinyGO analysis of the GC vs. AT content of the genes encoding the DEPs compared to the genes encoding all quantified proteins in the dataset. This revealed a significant shift (*p* = 0.002) towards higher GC content in genes encoding downregulated DEPs in U87_MUT_ vs. U87_WT_ (Figure 1C). This would conform to a higher probability of silencing methylations at CpG islands in U87_MUT_ than in U87_WT_ due to inhibited activity of 2OG-dependent TET demethylases. We found a similar shift towards higher GC content in genes encoding upregulated DEPs in PD-AS vs. PD-GB (Figure 1B). This cannot be explained by DNA hypermethylation. However, RNA methylation status is also regulated by 2OG-dependent demethylases. Of these, ALKBH5 and FTO are known erasers of m^6^A, the most abundant modification in mRNA [19,20], and they have also been assigned prognostic value in gliomas [21]. Their inhibition by 2OHG would lead to elevated levels of m^6^A in mRNA and may alter mRNA stability and translation efficiency depending on which “reader” proteins bind to the modifications [22,23,24]. Increased degradation of hypermethylated, adenine-rich transcripts could thus contribute to the shift in GC-content in the upregulated DEPs in PD-AS vs. PD-GB.

Among proteins commonly assigned prognostic/therapeutic value in glioma, several were differentially expressed in PD-AS vs. PD-GB (Figure 1D). Of these, PTEN was robustly detected in all PD-AS replicates but in neither of the PD-GB replicates. It is a well-established tumor suppressor that acts as a critical negative regulator of the PI3K signaling pathway, which is essential for controlling cell proliferation and survival. PTEN mutation (chromosome 10 loss) occurs frequently in glioblastoma and contributes to an immunosuppressive environment and resistance to immune checkpoint inhibitors [25].

CDKN2A (tumor suppressor ARF) was also strongly upregulated in PD-AS vs. PD-GB. Loss of CDKN2A is associated with shorter survival in both IDH mutant and wild-type gliomas [26]. EGFR was fourfold upregulated in PD-AS vs. PD-GB. Amplification or gain-of-function mutations in EGFR occur in about half of IDH_WT_ glioblastomas [27]. Whereas EGFR inhibitors have proven successful in e.g., lung cancer treatment, results have been disappointing in the treatment of gliomas. MGMT was threefold higher in PD-AS vs. PD-GB. It is the primary enzyme for the direct reversal of O6-methylguanosine, the most cytotoxic DNA lesion induced by TMZ, and *MGMT* promoter methylation status is an important prognostic biomarker and potential predictor of response to TMZ [28]. The uncertainty regarding predicting TMZ response is primarily due to discordance between analyses of promoter methylation status and MGMT expression, underscoring the importance of quantifying this biomarker at the protein level. TERT was below the detection levels in both PD-AS and PD-GB, indicating the absence of activating mutations in the TERT promoter. Finally, ATRX was robustly detected in both samples. However, given the plethora of *ATRX* mutations reported in gliomas [29], we cannot exclude the presence of functionally inactivating mutations.

We identified 84 DEPs that were common in our two IDH1 MUT/WT cell models, 25 of which were upregulated, and 59 of which were downregulated. Among the common downregulated DEPs in the IDH1 mutant cells, nearly half have previously been assigned potential value as prognostic/predictive biomarkers in gliomas, and of these, most predict a negative prognosis when highly expressed (Figure 1E, blue box, Supplementary Table S3). Interestingly, 12 of the common upregulated proteins in the IDH mutant cells have previously been associated with a negative prognosis, whereas only one, GNG2, has been associated with a positive prognosis. RB1 and PML were robustly detected in both PD-AS and U87_MUT_ but were below the level of detection in their IDH1 WT counterparts. RB1 is a tumor suppressor protein that, in its unphosphorylated form, binds E2F and halts the cell cycle at the G1/S phase border. Sequential phosphorylation by cyclinD-CDK4/6 and cyclin E-CDK2 promotes detachment of RB1 from E2F and allows S-phase entry. We did not assess the phosphorylation stoichiometry of RB1; however, cyclin D (CCND) and cyclin E (CCNE) were below the detection level in both cell models. Moreover, both CDK6 and CDK2 were significantly downregulated in PD-AS vs. PD-GB, whereas the cyclinD-CDK4/6 inhibitor CDKN2A was strongly upregulated. These alterations suggest enhanced RB1-induced arrest at the G1/S border in the PD-AS cells. Cell cycle analysis revealed, however, no G1/S-arrest, but rather accumulation of cells in G2 (Figure 1F, upper right panel). Additionally, a significant population of haploid or near-haploid cells was observed. This population does not appear to consist of apoptotic cell fragments, as mass spectrometry analysis showed no increase in activating CASP3 cleavages at Asp28 or Asp175 in PD-AS relative to PD-GB (Supplementary Figure S1). The underlying mechanism for this phenomenon remains unclear, but haploid cell clones have also been observed in other malignancies, including brain tumors [30,31]. Potential explanations include dysregulation of the G2/M or intra-M checkpoints, or downregulation of proteins associated with centrosome function or spindle assembly. Notably, whereas most proteins involved in the latter processes were expressed at similar levels in PD-AS and PD-GB, the outer kinetochore complex subunit KNL1 constituted an exception to this. It was robustly detected in all PD-GB replicates but was non-detectable in PD-AS. KNL1 is a large, predominantly unstructured protein that plays a key role in kinetochore-microtubule attachment and in early mitosis signaling [32,33], and defects in KNL1 are linked to primary microcephaly [34]. Although KNL1 is upregulated in a wide range of cancers, including gliomas [35], there is currently little evidence of a causal link between dysregulated KNL1 and tumorigenesis. An alternative explanation for the accumulation of apparently haploid cells in PD-AS could be upregulation of meiotic proteins. Genes that contribute to this belong to a class denoted cancer/testis genes, which are expressed in some tumors, including brain tumors [36]. We aim to study this further by single-cell transcriptome analysis of the PD-AS cells.

Finally, we compared the differentially expressed proteins (DEPs) between our IDH1_MUT/WT_ models and a recently published 36-gene panel (UAB36), in which increased mRNA expression correlates with resistance to over 20 cancer drugs across 777 cancer cell lines [37]. This gene set shows particularly strong associations with resistance to chemotherapeutics commonly used in glioblastoma treatment, including TMZ and carmustine. As illustrated in Figure 1G, most proteins encoded by these resistance-associated mRNAs were upregulated in PD-AS relative to PD-GB, whereas most of these proteins were downregulated in U87_MUT_ compared to U87_WT_ cells. Notably, the two common downregulated proteins in our two IDH mutant cell models, KRT8 and ABCC3, were recently assigned oncogenic roles in gliomas, and the low expression of these proteins predicted enhanced response to chemotherapy [38,39].

### 2.2. IDH Mutant Glioma Cells Display Elevated MHC Antigen Presentation and Interferon Signaling

**Figure 2:**
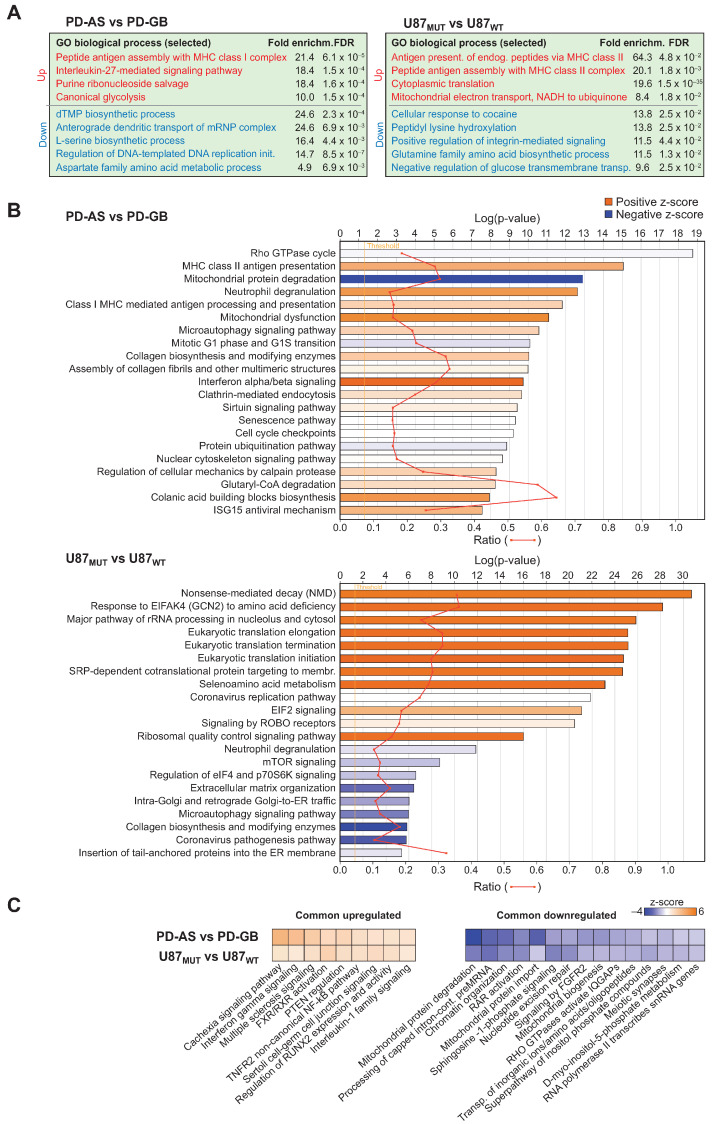
Analysis of differentially regulated biological processes in PD-AS vs. PD-GB and in U87_MUT_ vs. U87_WT_. (**A**) Most affected GO biological processes reported by the PANTHER over-representation test. (**B**) Most affected biological processes reported by IPA. (**C**) Most significantly, common upregulated (left panel) and downregulated (right panel) processes in the cell models.

Further mapping of the DEPs on GO biological processes using the PANTHER over-representation test revealed significant upregulation of processes such as antigen processing and assembly with MHC class I complex, purine ribonucleoside salvage, IL-27-mediated signaling, and canonical glycolysis as top differential biological processes in PD-AS vs. PD-GB (Figure 2A, left panel). Notably, in U87_MUT_ vs. U87_WT_, antigen presentation of endogenous peptides via MHC class II emerged as the most enriched pathway (Figure 2A, right panel). This shared emphasis on antigen presentation suggests a potential link between increased antigen presentation and the IDH1 mutation status. IPA analysis further supported these findings, identifying MHC class I and II antigen presentation as among the most upregulated pathways in PD-AS vs. PD-GB (Figure 2B, upper panel). Specifically, core components of the MHC I complex, HLA-A, HLA-B, and beta-2-microglobulin (B2M) were >100-, 68-, and 5.8-fold upregulated, respectively. This aligns with previous studies demonstrating reduced MHC I expression in migrating glioma cells, which helps these cells evade immune surveillance [40]. Furthermore, PD-AS exhibited a shift toward immunoproteasomes, which enhance MHC I antigen presentation by replacing canonical proteasomal β subunits (PSMB5/6/7) with interferon-γ-induced subunits (PSMB8/9/10). Consistent with this, PSMB8, PSMB9, and PSMB10 were upregulated by 4.1-, 9.2-, and 4.1-fold, respectively, in PD-AS vs. PD-GB, while the canonical subunits PSMB5 and PSMB6 were downregulated by 2.3- and 2.9-fold (Supplementary Table S1). In U87_MUT_ vs. U87_WT_, MHC-related upregulation was less pronounced compared to PD-AS vs. PD-GB. However, significant upregulation was observed in MHC II-related components, including HLA-DQA1, HLA-DRA, and HLA-DRB1. Notably, GO cellular component analysis of upregulated DEPs in U87_MUT_ vs. U87_WT_ highlighted significant enrichment of lysosomal proteins (*p* = 2.9 × 10^−5^), consistent with the lysosomal processing of internalized proteins for MHC II presentation.

The PANTHER analysis identified IL27 signaling as the second most upregulated pathway in PD-AS vs. PD-GB. IL-27 has previously been shown to mediate potent antitumor activity, with reduced IL-27 levels linked to increased glioma susceptibility [41]. Although IL-27 itself was not detected in our LFQ analyses, key downstream effectors within this pathway were markedly upregulated in PD-AS, including OAS1 (27-fold), OAS2 (>100-fold), MX1 (>100-fold), and STAT1 (6.2-fold). It should be noted, however, that several of these proteins are also upregulated by interferon and other cytokines, as corroborated by IPA analysis, which highlighted interferon α/β signaling and overall interferon responses as significantly enriched pathways in PD-AS (Figure 2B). These findings suggest a diminished inflammatory response in PD-GB compared to PD-AS. Supporting this, 13 of 15 canonical interferon-stimulated proteins quantified in our study were significantly upregulated in PD-AS, with some showing over 100-fold increases (e.g., IFIT1, IFIT2). Several of these proteins, such as IFI35 [42] and MX2 [43], have been proposed as therapeutic targets in glioma. Low IFIT2 expression, previously linked to PTEN loss, aligns with the lack of PTEN expression in glioblastoma tumors, which are also characterized by reduced CD8+ T-cell infiltration and impaired cGAS-STING signaling [14]. Consistently, STING1 was robustly expressed in PTEN-proficient PD-AS cells but below the detection limit in PTEN-deficient PD-GB cells (Supplementary Table S1). It should also be noted that IPA reported interferon gamma signaling as the most significantly common upregulated pathway in both PD-AS and U87_MUT_ (Figure 2C). Interestingly, recent studies have shown that elevated interferon signaling, including upregulation of STAT1, correlates with poor survival in glioblastoma patients and may represent a potential therapeutic target [44,45]. The significantly higher expression of STAT1 (6-fold, *p* = 8.5 × 10^−5^) and other interferon-stimulated genes in PD-AS cells compared to PD-GB highlights the relevance of further investigating these pathways, particularly in certain lower-grade gliomas.

Both PANTHER and IPA reported processes related to cytoplasmic translation among the most upregulated processes in U87_MUT_ vs. U87_WT_ (Figure 2A,B). It is tempting to speculate that this may, at least in part, be related to the strong (>100-fold) upregulation of the lysine methyltransferase SMYD5. Two independent reports very recently described lysine trimethylation of the mature ribosomal subunit RPL40. This protein is expressed from *UBA52* as a ubiquitin-ribosomal fusion protein that is subsequently cleaved into ubiquitin and mature RPL40. SMYD5-mediated trimethylation of K22 in RPL40 leads to a robust overall increase in translational output. Inhibiting the SMYD5-RPL40me3 axis reprograms protein translation to attenuate oncogenic gene expression signatures and renders tumor cells hypersensitive to mTOR inhibitors [46,47]. To what degree RPL40me3 is a reversible modification remains elusive. In histone H3, however, K9me3 embedded in an RKS sequence is demethylated by 2OG-dependent PHF8, which is strikingly similar to the RKC sequence at K22me3 in RPL40.

Paradoxically, IPA reported collagen biosynthesis and modifying enzymes among the top upregulated pathways in PD-AS vs. PD-GB, whereas this pathway was among the most downregulated in U87_MUT_ vs. U87_WT_ (Figure 2B). In PD-AS, upregulation was especially evident for collagen II, IV, and VI, as well as the 2OG-dependent prolyl- and lysyl hydroxylases critical for their maturation. In contrast, nearly all these proteins were significantly reduced in U87_MUT_ to U87_WT_ (Supplementary Table S1). Although 2OHG is expected to inhibit collagen hydroxylases, potentially causing misfolded collagen accumulation and ER stress, we detected no broad ER stress response in either IDH-mutant model. The sole exception was significantly higher SERPINH1 (HSP47) levels in both cell types. Indeed, in PD-AS, SERPINH1 ranked second among all proteins by MS intensity (Supplementary Table S1). Furthermore, the collagen prolyl-4-hydroxylase (P4H) subunit P4HA1 was sixfold upregulated in PD-AS, whereas the subunit P4HB was the fourth most abundant among the PD-AS proteins. This suggests that a high abundance of collagen P4H may partially overcome 2-OHG’s inhibitory effects, maintaining sufficient collagen hydroxylation. Additionally, SERPINH1, a specialized collagen-binding chaperone, not only facilitates procollagen folding and ER-to-Golgi transport independent of collagen P4H activity, but may also stabilize under-hydroxylated collagen, preventing aggregation [48].

### 2.3. IDH1 Mutations Are Associated with Distinct Metabolic Shifts Depending on Genetic Background

Purine salvage was among the top upregulated pathways in PD-AS vs. PD-GB, with significant upregulation of S-methyl-5-thioadenosine phosphorylase (MTAP, 3.3-fold), adenosine kinase (ADK, 3.2-fold), purine nucleoside phosphorylase (PNP, 3.2-fold), adenine phosphoribosyltransferase (APRT, 2.1-fold), and phosphopentomutase (PGM2, 1.8-fold). Notably, loss of MTAP expression occurs in about 45% of GBs, and is accompanied by increased stemness and more aggressive tumors [49]. MTAP is a key enzyme in the purine and methionine salvage pathways. It catalyzes the breakdown of methylthioadenosine, a byproduct of polyamine biosynthesis, into adenine and methionine, thereby salvaging these metabolites for other biochemical reactions. Loss of MTAP results in epigenetic dysregulation in glioma cells, and inhibition of de novo purine biosynthesis was recently shown to specifically deplete therapy-resistant GB stem-cell-like cells [49]. Neither of the above purine salvage enzymes was upregulated in U87_MUT_ vs. U87_WT_, suggesting that the altered purine metabolism is not an obligatory consequence of IDH1 mutation. Preclinical studies recently suggested that targeting inosine-5′-monophosphate dehydrogenase (IMPDH), a key enzyme in the de novo purine pathway, can improve the efficacy of radiation and TMZ in GB. A follow-up phase 0/1 clinical study very recently demonstrated that the IMPDH inhibitor Mycophenolate mofetil (MMF), a commonly used oral immunosuppressant, was reasonably well tolerated, and a randomized phase 2/3 trial through the Alliance for Clinical Trials in Oncology is currently being planned [50]. Our findings suggest that quantification of proteins in salvage purine biosynthesis could aid the stratification of glioma patients in such trials.

**Figure 3:**
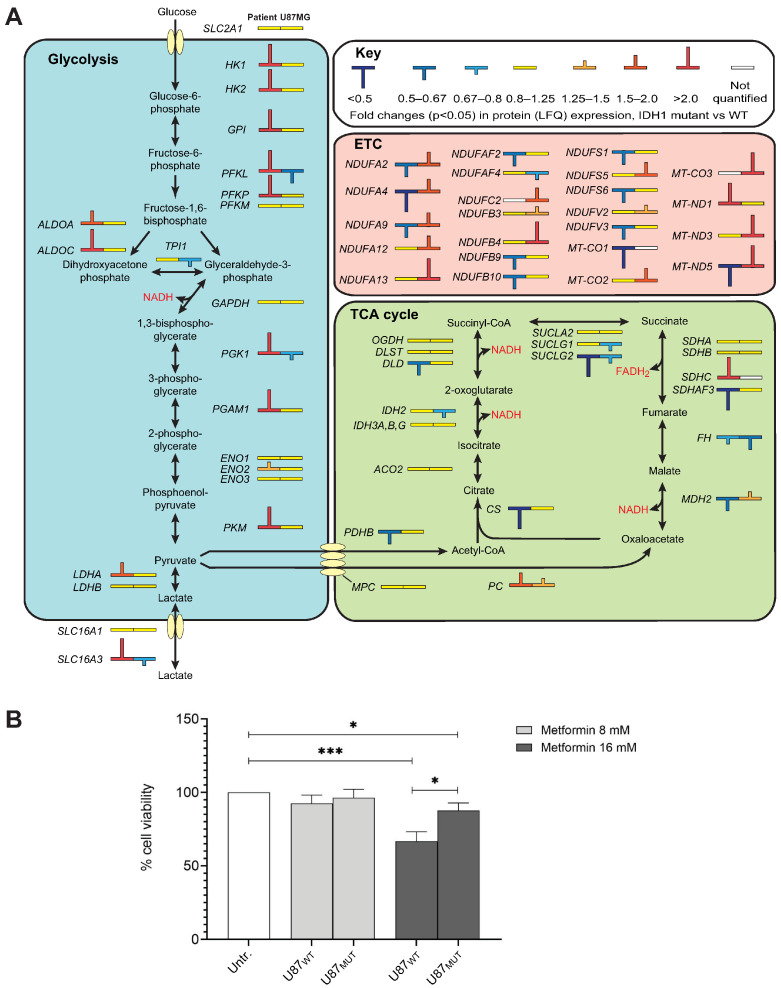
Opposite metabolic shifts and drug resistance profiles observed in PD-AS vs. PD-GB compared to U87_MUT_ vs. U87_WT_. (**A**) Whereas the IDH1-mutant PD-AS displayed a marked upregulation of glycolytic enzymes and downregulation of ETC proteins compared to PD-GB, the U87_MUT_ cells had downregulated or unchanged glycolytic enzymes and overall upregulated ETC proteins compared to U87_WT_. (**B**) The Complex I inhibitor metformin was significantly more cytotoxic to U87_WT_ than to U87_MUT_. Statistical significance was calculated using one-way ANOVA followed by Turkey post hoc test ( * *p* = 0.01–0.05; *** *p* = 0.0001–0.001).

PANTHER analysis also identified canonical glycolysis as one of the top upregulated pathways in PD-AS compared to PD-GB. This finding was corroborated by IPA analysis, which highlighted upregulated glycolysis (*p* = 8.6E-4, z-score 2.6) alongside downregulated TCA cycle activity (*p* = 1.2E-4, z-score −1.9) and oxidative phosphorylation (*p* = 5.2 × 10^−5^, z-score −2.5) in PD-AS relative to PD-GB. Notably, ten glycolytic enzymes were upregulated, whereas TCA cycle enzymes and proteins in the electron transport chain (ETC) were overall downregulated (Figure 3A). This metabolic reprogramming, characterized by a preference for glycolysis even in the presence of oxygen, is commonly referred to as the Warburg effect and is frequently associated with gliomas, as reviewed in [51]. Mutations in either IDH1 or IDH2 can contribute to this phenomenon via several mechanisms. First, in IDH1 mutant gliomas, cytosolic 2OHG may be transported into mitochondria, where it disrupts TCA cycle function and ATP production via oxidative phosphorylation [52]. Second, the balance between glycolysis and the TCA cycle is regulated by the transcription factor HIF1, which is key to cellular oxygen sensing. It is a heterodimer consisting of an unstable (HIF1A) and a stable (ARNT) subunit. At normoxia, HIF1A becomes hydroxylated by oxygen- and 2OG-dependent prolyl hydroxylases EGLN1,2 and 3, which mediates its proteasomal degradation. Under hypoxic conditions, hydroxylation is attenuated, leading to increased levels of HIF1A and a HIF1-mediated transcriptional response, including upregulation of several glycolytic enzymes and suppressed flow of carbon into the TCA cycle via activation of PDK1 [53]. Inhibition of the EGLNs by 2OHG would stabilize HIF1A even under normoxic conditions, leading to a “pseudohypoxic” state [54]. Third, 2OHG also attenuates the activity of the HIF1A-inhibitor HIF1AN, which hydroxylates HIF1A at Asn803 and blocks binding to its transcriptional coactivators [53]. Fourth, the high levels of collagen P4H and the abundance of substrate collagens in PD-AS likely drain 2OG and oxygen available for EGLN1–3, since collagen P4H has markedly higher affinity for both co-substrates than the HIF prolyl hydroxylases [55], and thereby contributes to HIF1 stabilization. Finally, factors not directly related to the IDH1 status may contribute to the glycolytic shift in PD-AS compared to PD-GB. One such candidate is EGFR, which was fourfold higher expressed in PD-AS vs. PD-GB, whereas it was not significantly different in U87_MUT_ vs. U87_WT_. EGFR signaling upregulates glycolytic enzymes and downregulates OXPHOS, and inhibitors targeting EGFR have been shown to reverse this process [56].

Interestingly, we observed no shift from TCA to glycolysis in U87_MUT_ cells compared to U87_WT_ cells, suggesting that induction of a pseudohypoxic state is not an obligatory consequence of mutated IDH1. Potentially, this could be mediated by differential expression of factors involved in HIF1 regulation and transcriptional output in the PD and U87 cell models. Notably, however, TCA activity does not solely depend on the levels of the involved proteins. Accumulating evidence suggests that the functions of several TCA cycle enzymes are regulated by binding to long non-coding RNAs (lncRNAs) [57]. One such example is the lncRNA GAS5, which binds MDH2 and thereby inhibits the metabolic enzyme tandem association of FH, MDH2, and CS [58]. Very recent research strongly indicates that the level of GAS5 is regulated by m^6^A modification in gliomas. Firstly, the levels of the m^6^A erasers FTO and ALKBH5 are very significantly negatively correlated with GAS5. Secondly, GAS5 expression is significantly higher in IDH mutant- than in IDH WT gliomas (*p* = 2.9 ×10^−18^) [59]. It is thus tempting to speculate that 2OHG-mediated inhibition of FTO and ALKBH5 downregulates TCA flux in U87_MUT_ via upregulation of GAS5.

Mitochondrial electron transport (NADH to ubiquinone) was among the most upregulated biological processes in U87_MUT_ (Figure 2A), driven by a notable increase in several Complex I components (Figure 3A). Potentially contributing to this could be that ETC proteins are upregulated by HIF1 under normoxia [60]. In U87_MUT_, the accumulation of 2OHG would inhibit HIF1 hydroxylation and inactivation, resulting in constitutive overexpression of HIF1-regulated genes. Enhanced ETC activity in U87_MUT_ was further supported by the fourfold upregulation of the mitochondrial glutathione transporter SLC25A40. Since glutathione is exclusively synthesized in the cytosol, its import into mitochondria via SLC25A39/40 is critical for the proper functioning of ETC proteins, particularly those containing iron-sulfur clusters [61], which are abundant in Complex I.

The upregulation of Complex I, an NADH-accepting component of the ETC, despite no apparent changes in glycolytic or TCA cycle activity, suggests alternative sources of NADH in U87_MUT_ cells. We hypothesize that fatty acid oxidation may serve as a major contributor to NADH production in this context. Supporting this, CPT1A, which catalyzes the rate-limiting step in β-oxidation by transferring the acyl group of long-chain fatty acid-CoA conjugates onto carnitine, was upregulated 1.6-fold (*p* = 0.02) in U87_MUT_ compared to U87_WT_. Previously, knockdown of CPT1A in an IDH1_WT_ background reduced tumor growth and increased survival in a glioma mouse xenograft model [62]. Interestingly, metformin, which is widely used to treat type 2 diabetes, is a reversible inhibitor of the ETC Complex I [63]. It is also one of the few drugs that can cross the blood–brain barrier [64]. A recent Phase I trial of combined metformin and radiation treatment for high-grade gliomas demonstrated promising clinical efficacy [65]. However, there was no information on the IDH status of the tumors. Likewise, a recruiting Phase II trial (NCT04945148) will include metformin in a standard radiation/TMZ treatment of newly diagnosed GB (IDH_WT_). Our results suggest that future research and clinical trials encompassing drugs that target fatty acid oxidation and the ETC should also encompass patients with IDH1_MUT_ gliomas.

To investigate whether inhibition of Complex I could be exploited as a specific vulnerability in gliomas with increased ETC and normal glycolytic flux, we treated U87_MUT_ and U87_WT_ with metformin, which has been suggested as a potentially selective adjuvant in the treatment of IDH-mutant gliomas [66]. Somewhat unexpectedly, the U87_WT_ cells were significantly more sensitive to 16 mM metformin than U87_MUT_ (Figure 3B). The most logical explanation for this would be that the U87_MUT_ cells harbor enhanced levels of functional Complex I and thus tolerate higher levels of the inhibitor. It will be interesting in the future to study how metformin affects these cells when co-administered with other metabolic inhibitors, e.g., glutaminase inhibitors.

Collectively, our findings indicate that the metabolic alterations associated with IDH1 mutations in glioma cells strongly depend on the overall genetic background of the cells. Potentially, the U87_MUT_ cells have elevated compensatory mechanisms to buffer cytosolic 2OG, compared to PD-AS. Such mechanisms could include elevated mitochondrial 2OG generation by enhanced TCA cycle turnover or by decreased OGDH expression [67], increased 2OG transport from mitochondria to the cytoplasm, or increased glutaminolysis. In support of this, TCA enzymes were more negatively affected by the IDH1 mutation in PD-AS than in U87_MUT_ (Figure 3A). Moreover, U87_MUT_ had higher levels of the mitochondrial 2-oxoglutarate/malate carrier SLC24A11 and glutamate dehydrogenase (GLUD1) than PD-AS, based on normalized MS intensity values. Finally, the nature of the IDH1 mutant likely contributes to higher 2OHG in PD-AS than in U87_MUT_, since the R132H mutant (U87_MUT_) has a lower 2OHG production capacity than R132C (PD-AS) [68].

### 2.4. DNA Repair Processes Are Differentially Affected in IDH-Mutant Glioma Cells

DNA repair is essential for maintaining genomic stability in cells, and mapping repair activity can help predict tumor responses to DNA-damaging chemotherapeutic agents and radiation [69]. The impact of IDH mutations on DNA repair has largely been attributed to 2OHG-mediated inhibition of DNA and histone demethylation via TET enzymes and KDMs. Many DNA repair proteins are regulated by promoter methylation, which can influence treatment strategies. Additionally, DNA hypermethylation may contribute to a mutator phenotype, as the spontaneous deamination of 5-methylcytosine (5-meC) occurs at a significantly higher rate than that of unmethylated cytosine, leading to C > T mutations if not properly repaired [70]. However, IDH1 R132H mutations have also been proposed to act as tumor suppressors in gliomas by epigenetically upregulating mRNAs encoding DNA damage response (DDR) proteins [71].

**Figure 4:**
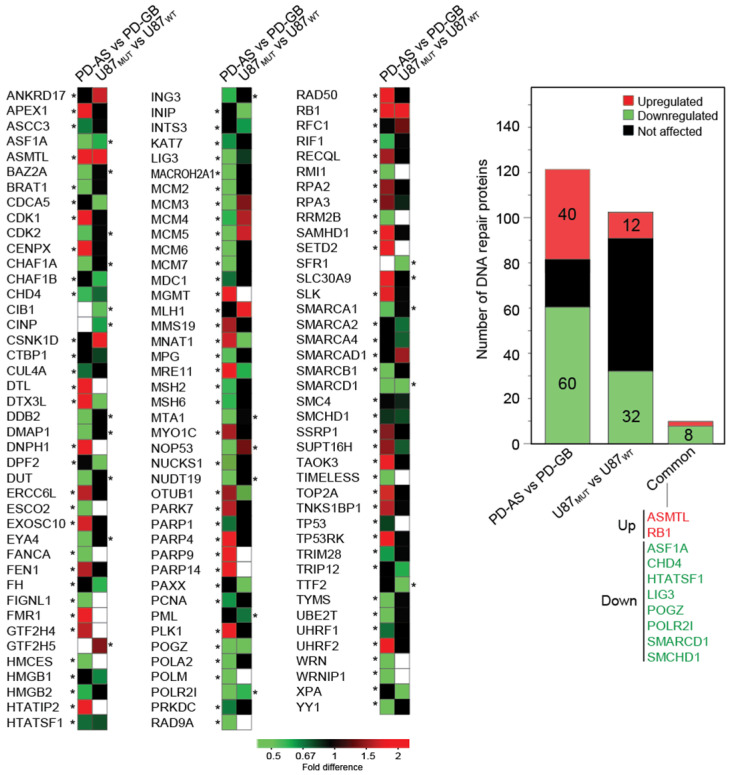
Most DEPs associated with DNA repair and genome maintenance are downregulated in IDH1 mutant glioma cells (PD-AS and U87MUT) compared to IDH1 wild-type glioma cells (PD-GB and U87_WT_). Graphic representation of DNA repair proteins significantly differentially expressed (> + / − 1.5-fold, *p*  <  0.05) in at least one of the IDH1 mutants (PD-AS or U87_MUT_) is shown in the left panel. Asterisks denote which of the two IDH1 mutants has the highest baseline expression of each protein. The common ten DEPs found in both cell models are shown in the right panel.

To explore whether this regulation extends to protein expression, we analyzed our LFQ data for proteins implicated in DNA repair and genome maintenance [72,73,74]. Among the 98 DNA repair proteins that were differentially expressed in at least one dataset (Figure 4, left panel), 30 were significantly upregulated, while 51 were significantly downregulated in PD-AS vs. PD-GB. Similarly, 11 proteins were upregulated and 23 were downregulated in U87_MUT_ vs. U87_WT_ (Figure 4, right panel). Notably, both IDH-mutant cell models exhibited a predominance of downregulated DNA repair-associated DEPs compared to their wild-type counterparts. This highlights the poor correlation between mRNA and protein levels, particularly in tumor cells [6]. The smaller number of DNA repair DEPs in U87_MUT_ compared to the isogenic U87_WT_ suggests that the differential expression of DNA repair proteins in PD-AS vs. PD-GB is primarily driven by other factors than the IDH1 mutant. This aligns with the small overlap in total DEPs observed between the two IDH_MUT_/_WT_ models (Figure 1E).

Among the proteins involved in DNA repair and genome maintenance, RB1 and ASMTL were the only two commonly upregulated (>100-fold) proteins across both IDH_MUT_ models compared to their IDH_WT_ counterparts. Beyond its well-established role in G1/S checkpoint regulation, RB1 also plays a role in the repair of DNA double-strand breaks (DSBs) that is independent of cell cycle regulation, and loss of RB1 sensitizes prostate cancer cells to ionizing radiation [75]. Paradoxically, loss of RB1 expression has been reported as a negative prognostic factor in several neoplasms, including gliomas [76,77,78]. This suggests that increased mutation rates due to the loss of RB1-mediated G1/S checkpoint control may outweigh the reduced DNA repair capacity in determining tumor fitness, especially during treatment with genotoxic agents. ASMTL is a member of the Maf (multicopy-associated filamentation) family of evolutionarily conserved “house-cleaning” nucleotide pyrophosphatases. These enzymes hydrolyze a variety of canonical and non-canonical nucleoside triphosphates into their corresponding monophosphates and are thought to play critical roles in both cell cycle arrest and the prevention of modified nucleotide incorporation into cellular nucleic acids. Human ASMTL exhibits its highest enzymatic activity against canonical nucleotides such as UTP, CTP, dTTP, and dCTP, as well as non-canonical nucleotides, including pseudo-UTP, 5-methyl-UTP, 5-methyl-CTP, N4-methyl-dCTP, 8-oxo-GTP, and N1-methyl-GTP [79]. The extent to which the altered metabolic state of IDH1-mutated glioma cells contributes to the markedly enhanced expression of ASMTL remains unclear. However, it is plausible that elevated levels of 8-oxo-GTP, which accumulate due to the increased oxidative stress characteristic of IDH1-mutated malignancies, could act as a contributing factor. Notably, 8-oxo-GTP can serve as a substrate for RNA polymerase II during transcription, thereby impairing translation and causing erroneous protein synthesis [80,81].

The human NUDT1 (MTH1) enzyme is also capable of hydrolyzing 8-oxo-GTP to 8-oxo-GMP, albeit with an efficiency approximately 50-fold lower than its activity in hydrolyzing 8-oxo-dGTP [81]. Consequently, NUDT1 is primarily regarded as a key enzyme for clearing oxidized deoxyribonucleotides from the nucleotide pool. Since ASMTL and NUDT1 exhibited similar expression levels in both IDHMUT/WT models, ASMTL is likely the primary enzyme responsible for clearing 8-oxo-GTP in these glioma cells. Potentially, ASMTL-mediated clearance of 8-oxo-GTP may also contribute to the resistance to oxidative stress induced by chemotherapy, including TMZ [82]. A growing body of evidence, including work from our own laboratory, suggests that chemotherapy-induced damage to RNA may play a more prominent role in cytotoxicity than previously anticipated [83,84,85,86].

Interestingly, of the eight DNA repair proteins commonly downregulated in our two IDH1_MUT_ cell models (Figure 4, right panel), seven have been implicated in the repair of DSBs, primarily via homologous recombination (HR). The replication-dependent histone chaperone ASF1A promotes HR repair of DSBs at stalled or collapsed replication forks [87]. CHD4 is part of the histone deacetylase NuRD complex and promotes HR repair via upregulation of RAD51 in glioma cells [88], while HTATSF1 recruits TOPBP1 to PARylated RPA to facilitate RPA/RAD51-dependent HR [89]. POGZ recruits heterochromatin proteins to maintain BRCA1/BARD1 at DSBs and promote HR [90]. Two of the downregulated proteins, SMCHD1 and SMARCD1, are parts of chromatin remodeling complexes that promote DSB repair via NHEJ [90,91].

Finally, it should be noted that one of the interferon-induced proteins that was highly upregulated in PD-AS vs. PD-GB, SLFN11 (96-fold, *p* = 0.0001), acts as a negative regulator of HR [92], and that high expression of SLFN11 shows an extremely significant positive correlation with the response to TOP1/2 inhibitors, alkylating agents and DNA synthesis inhibitors in in cancer cell lines of different origin [93]. Nevertheless, the Human Protein Atlas (www.proteinatlas.org, accessed on 20 February 2025) reports high expression of SLFN11 mRNA as a poor prognostic factor in glioblastoma (*p* < 0.001). The underlying reasons for this apparent discrepancy remain, however, to be elucidated.

### 2.5. Potential Contribution of Altered Histone Lysine Methylation to DNA Damage Responses in the IDH-Mutant Cell Models

**Figure 5:**
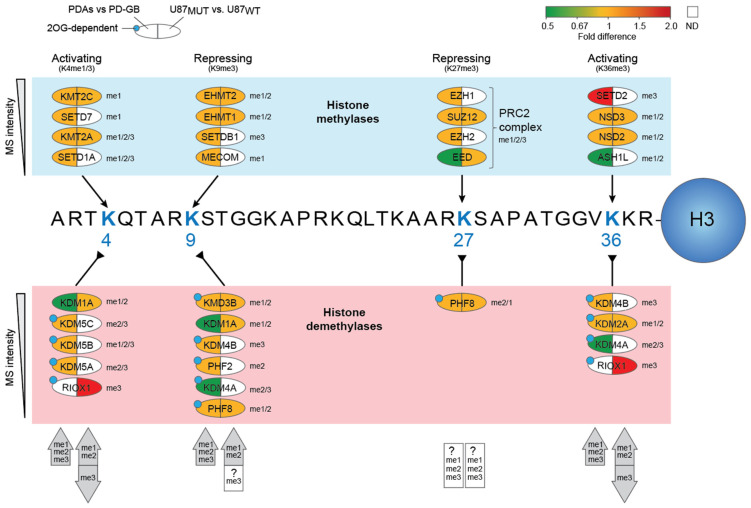
The IDH1-mutant glioma cells harbor distinct expression of proteins regulating histone lysine methylation. Histone lysine methyltransferases (KMTs) detected in our datasets are shown in the blue box (ranked after MS intensities in PD-AS) and pointing to their respective lysine targets in Histone H3. Likewise, histone lysine demethylases (KDMs) are shown in the pink box, below their lysine targets. A filled blue circle indicates that the KDM is 2OG-dependent and thus should be inhibited in the IDH1-mutant cells. The color bar at the top right indicates fold change in expression in the IDH-mutant cells vs. their IDH wild-type counterparts.

The efficiency of DNA damage responses is determined not only by the levels and activities of DNA repair proteins but also by their accessibility to DNA lesions within chromatin. This accessibility is dynamically regulated by post-translational histone modifications, including methylation. Additionally, histone methylation directly or indirectly influences the transcription of many DNA repair genes. Particularly in the context of DSB repair, histone methylations dictate the choice between HR or NHEJ pathways [94]. Histone methylation is controlled by histone lysine methyltransferases (KMTs) and histone lysine demethylases (KDMs) and can either activate or repress gene expression, depending on the specific residues modified and the number of methyl groups added. Dysregulated histone methylation has been implicated in various cancers, including gliomas [95]. Among the most well-characterized histone methylation marks are trimethylation at lysine residues K4, K9, K27, and K36 on histone H3. Of these, H3K4me3 and H3K36me3 are generally associated with active gene expression, whereas H3K9me3 and H3K27me3 typically mediate transcriptional repression. We quantified 16 KMTs and 15 KDMs across our cell models, including ten 2OG-dependent demethylases (Figure 5, Supplementary Table S4).

Based on the expression levels of involved proteins, distinct and complex alterations of histone lysine methylation in the IDH1 mutants emerge compared to their IDH1 WT counterparts. Our findings suggest that the IDH1-mutant PD-AS model exhibits increased mono- di- and trimethylation at H3K4, H3K9, and H3K36 compared to the IDH1 wild-type PD-GB model. In particular, H3K36me3 is likely to be highly enriched in PD-AS vs. PD-GB due to the marked upregulation (>100-fold) of SETD2, the sole human methyltransferase responsible for catalyzing this modification. This effect is further reinforced by 2OG-mediated inhibition of the H3K36me3 demethylases KDM4A/B and the concomitant four-fold downregulation of KDM4A. Depletion of SETD2 has been shown to severely block HR repair [96]. Thus, although many HR proteins were downregulated in PD-AS vs. PD-GB, the marked upregulation of SETD2 may compensate for this and allow efficient repair of HR in actively transcribed genes. Conversely, since SETD2 was below the detection limit in U87, this should further disfavor HR. Notably, however, it appears that inactive genes depleted in H3K36me3 may instead be targeted to NHEJ repair [97].

SETD2-induced H3K36me3 also plays a crucial role in recruiting the DNA mismatch repair (MMR) machinery to chromatin via interaction with MutSα (MSH2/MSH6) [98]. This may be especially important in the context of mutated IDH, since this would enhance genomic 5-meC due to TET inhibition, leading to enhanced levels of 5-meC deamination-induced G:T mismatches, which are repaired by MMR [99]. Since SETD2 remained below the detection level in U87_MUT_, we speculate that the marked induction in PD-AS is not directly mediated by the IDH1 mutation. Nevertheless, it may be of strong predictive value regarding the treatment response to TMZ. Intact MMR promotes TMZ cytotoxicity, especially in MGMT-deficient gliomas, since TMZ-induced O6-meG mispairs with thymine. During MMR, O6-meG persists while thymine is removed, leading to thymine reinsertion and cytotoxic futile repair cycles. This leads to gradually increased DNA resection, induction of SSBs and DSBs, and apoptosis [100]. Notably, both MSH2 and MSH3 were significantly reduced in PD-AS (Figure 4). Nevertheless, MMR may still be enhanced compared to PD-GB due to increased targeting to the mismatches via H3K36me3.

We hypothesize that the opposite regulation of trimethylation at H3K36 also holds true for H3K4. Firstly, SETD1A, a key H3K4me3 methyltransferase, remained below the detection level in U87_MUT_ vs. U87_WT_. In addition, the >100-fold upregulation of RIOX1, which also catalyzes me3 demethylation at this position, should further contribute to H3K4me3 depletion. Since HIF1-α prefers H3K4me3-decorated binding sites [101], primarily associated with promoters and transcriptional start sites [102], this might contribute to the different metabolic alterations observed in the two IDH_MUT_ cell models. Notably, the proposed inverse level of H3K4me3 and H3K36me3 in our two IDH_MUT_ models is also corroborated by the ratio between up- and downregulated DEPs (Figure 1B), in which there is a marked shift towards downregulated DEPs in U87_MUT_ vs. U87_WT_.

## 3. Materials and Methods

### 3.1. Cell Culture

Isogenic IDH1 wild-type (U-87MG HTB-14) and heterozygous mutant (R132H) (U-87MG HTB-14IG) were purchased from ATCC (Manassas, VA, USA). The cells were grown in 5% CO_2_ at 37 °C, in DMEM high glucose medium (Sigma-Aldrich, St. Louis, MO, USA) supplemented with 10% fetal bovine serum (FBS, Sigma-Aldrich, St. Louis, MO, USA), 100 µg/mL penicillin-streptomycin (P/S, Sigma-Aldrich, St. Louis, MO, USA), and 2 mM L-glutamine (Sigma-Aldrich, St. Louis, MO, USA). The patient-derived cells (PD-AS, PD-GB) were cultured on Geltrex-coated plates (Gibco, Waltham, MA, USA) in 5% CO_2_ at 37 °C, in Knockout DMEM/F12 (Gibco, Waltham, MA, USA) medium supplemented with 1×Stempro Neural Supplement (Gibco, Waltham, MA, USA), 2 mM L-glutamine (Sigma-Aldrich, St. Louis, MO, USA), 100 µg/mL P/S (Sigma-Aldrich, St. Louis, MO, USA), and 20 ng/mL of both Fibroblast Growth Factor 2 (FGF2, Sigma-Aldrich, St. Louis, MO, USA) and Epidermal Growth Factor (EGF, Sigma-Aldrich, St. Louis, MO, USA). ROCK Inhibitor (Sigma-Aldrich, St. Louis, MO, USA) was added to the culture medium for the first 24 h following passaging to ensure a single-cell suspension.

### 3.2. Cell Cycle Analyses

Cells were fixed in ice-cold methanol at a concentration of 10^4^–10^5^ cells/mL. After fixation, cells were washed with PBS and incubated with 200 µL RNase A solution (100 µg/mL in PBS) at 37 °C for 30 min. DNA was stained by adding 200 µL of propidium iodide (PI, 50 μg/mL, Sigma, St. Louis, MO, USA), followed by incubation at 37 °C for 30 min. Flow cytometry was performed using a BD FACS Canto flow cytometer (BD Biosciences, San Jose, CA, USA), with PI fluorescence excited at 488 nm (blue laser) and detected in the phycoerythrin (PE) channel (578 nm). Data were analyzed using FlowJo v10 software.

### 3.3. Viability Assay

A total of 5000 U87 cells per well were seeded in 96-well plates. After 24h incubation at 37 °C, cells were treated with Metformin or DMSO vehicle and incubated further for 72 h. To determine cell viability, a PrestoBlue assay was conducted. The PrestoBlue^TM^ HS Cell Viability Reagent (Thermo Fischer, Waltham, MA, USA, P50201) was prepared in culture medium according to the manufacturer’s instructions. After 30 min incubation at 37 °C, fluorescence intensity was measured using a BMG-Fluostar Omega plate reader (BMG Labtech, Ortenberg, Germany). Each treatment was performed in triplicate and in two independent experiments. The data were normalized to the vehicle-treated control (DMSO) (Sigma-Aldrich, St. Louis, MO, USA) and plotted using GraphPad Prism 10.

### 3.4. LC-MS/MS Analyses

Cells were cultured in 10 cm plates and incubated at 37 °C until a confluency of approximately 50%. Cells were harvested by trypsination and resuspended in 100 µL 1% sodium deoxycholate, 100 mM Tris-HCl, pH 8.5, 10 mM Tris (2-carboxyethyl)phosphine (TCEP), 40 mM chloroacetamide (CAA), and heated at 90 °C for 45 min. After sonication for 10 cycles (30 s ON/30 s OFF) with a Bioruptor^®^ Pico sonicator (Diagenode, Liège, Belgium), debris was pelleted at 16,000× *g* for 10 min. 50 µg protein from each sample was added to 100 µL 0.1 M ammonium bicarbonate, 0.5 µg trypsin, and digested at 37 °C overnight. Peptides were desalted using C18 spin columns, dried in a speedvac centrifuge, and resuspended in 0.1% formic acid (FA) prior to MS analysis. LC-MS/MS was performed on a timsTOF Pro (Bruker Daltonics, Bremen, Germany) connected to a nanoElute (Bruker Daltonics, Bremen, Germany) HPLC. Five hundred ng peptides from each sample were injected and separated over a Bruker15 (75 µm × 15 cm) column with running buffers A (0.1% FA) and B (0.1% FA in acetonitrile) with a gradient from 2% B to 40% B over 100 min. The timsTof was operated in DDA PASEF mode with 10 PASEF scans per acquisition cycle and accumulation and ramp times of 100 ms each. The ‘target value’ was set to 20,000, and dynamic exclusion was activated and set to 0.4 min. The quadrupole isolation width was set to 2 Th for *m*/*z* < 700 and 3 Th for *m*/*z* > 800.

### 3.5. Bioinformatic Analyses

Raw data were inspected using Open Workflow [103] to determine optimal search parameters for MaxQuant v2.4.3.0 [104]. In addition to default settings, deamidation of asparagine and glutamine was included as a dynamic post-translational modification. Protein identification was performed using Andromeda, the built-in search engine of MaxQuant, against the human proteome, including isoforms, downloaded from UniProt (April 2023), along with MaxQuant’s internal contaminant database. To minimize missing values, the match-between-runs (MBR) function was enabled. MBR identifies peptide signals present in one sample but absent in another by leveraging high mass accuracy and a restricted retention time window of 1 min, within an overall 20 min sliding window. If a matching signal is detected, it is labeled as ‘By matching’ instead of ‘By MS/MS’ in the output. Protein and peptide group identifications were filtered to a 1% false discovery rate (FDR), and only high-confidence unique peptides were used for final protein group identification. LFQ [105] was performed using at least one unique peptide, followed by log_2_ transformation of LFQ values. The median value of biological replicates was used to represent the log_2_-LFQ value for each protein group. Statistical analyses were conducted using two-sided Student’s t-tests in R: (https://www.r-project.org/, accessed on 22 November 2024). *p*-values were computed, ranked, and adjusted for multiple testing using the Benjamini–Hochberg correction [106] to obtain an FDR-adjusted *p*-value (q-value). For proteins present in all control replicates but absent in all experimental samples, the LFQ ratio was set to −100 with an FDR of 0.0. Conversely, proteins detected in all experimental replicates but absent in controls were assigned an LFQ ratio of 100, with both *p*- and q-values set to 0.0. Biological pathway analyses were performed using the PANTHER overrepresentation test (Release 20221013, GO Ontology database, released 1 July 2022) [107,108], ShinyGO (http://bioinformatics.sdstate.edu/go/, accessed on 11 November 2024) [109], and Ingenuity^®^ Pathway Analysis (IPA) (Ingenuity Systems, www.ingenuity.com, Redwood City, CA, USA) [110].

## 4. Conclusions

Our findings underscore the complexity of predicting glioma pathogenesis and treatment responses based solely on IDH1 mutation status. While processes such as MHC antigen presentation and interferon signaling were consistently upregulated in our IDH1-mutant models, other pathways, particularly those involving metabolic reprogramming, varied significantly. This variability suggests that the phenotypic consequences of IDH1 mutations are not determined by the mutation in isolation but are instead shaped by the broader genomic and proteomic context, including the differences in expression of key glioma oncogenes and tumor suppressors that may modulate the impact of the 2OHG oncometabolite. These observations highlight the critical importance of investigating IDH1 mutations in experimental systems that represent a spectrum of genetic backgrounds to capture the range of potential biological outcomes and treatment vulnerabilities. Although proteomic profiling offers valuable insights, a comprehensive understanding of IDH1-mutant gliomas to aid treatment decisions will require integrative multi-omics approaches, including DNA/RNA methylation profiling, histone and protein post-translational modifications, and targeted DNA damage and repair assays.

## Data Availability

The mass spectrometry proteomics data have been deposited to the ProteomeXchange Consortium via the PRIDE [186] partner repository with the dataset identifier PXD065327.

## Supplementary Materials

The following supporting information can be downloaded at https://www.mdpi.com/article/10.3390/ijms26189075/s1. References [79,111,112,113,114,115,116,117,118,119,120,121,122,123,124,125,126,127,128,129,130,131,132,133,134,135,136,137,138,139,140,141,142,143,144,145,146,147,148,149,150,151,152,153,154,155,156,157,158,159,160,161,162,163,164,165,166,167,168,169,170,171,172,173,174,175,176,177,178,179,180,181,182,183,184,185] are cited in the Supplementary Materials.

## Abbreviations

The following abbreviations are used in this manuscript:

2OG	α-ketoglutarate, 2-oxoglutarate
2OHG	D-2-hydroxyglutarate
DEP	Differentially expressed protein
ETC	Electron transport chain
GO	Gene ontology
HLA	Human leukocyte antigen
IDH	Isocitrate dehydrogenase
KMT	Lysine methyltransferase
KDM	Lysine demethylase
LC-MS/MS	Liquid chromatography with tandem mass spectrometry
LFQ	Label-free quantitative
MHC	Major histocompatibility complex
PD-AS	Patient-derived astrocytoma, IDH1 mutant
PD-GB	Patient-derived glioblastoma, IDH wild-type
TMZ	Temozolomide
