# Supplementary material for: Proteomic Analysis Uncovers Enhanced Inflammatory Phenotype and Distinct Metabolic Changes in IDH1 Mutant Glioma Cells
Source: Int J Mol Sci. 2025 Sep 18;26(18):9075. doi: 10.3390/ijms26189075 (PMC12470816; doi:10.3390/ijms26189075)
Supplement: Supplementary file 1 [file ijms-26-09075-s001.zip › Supplementary Figure S1.pdf]

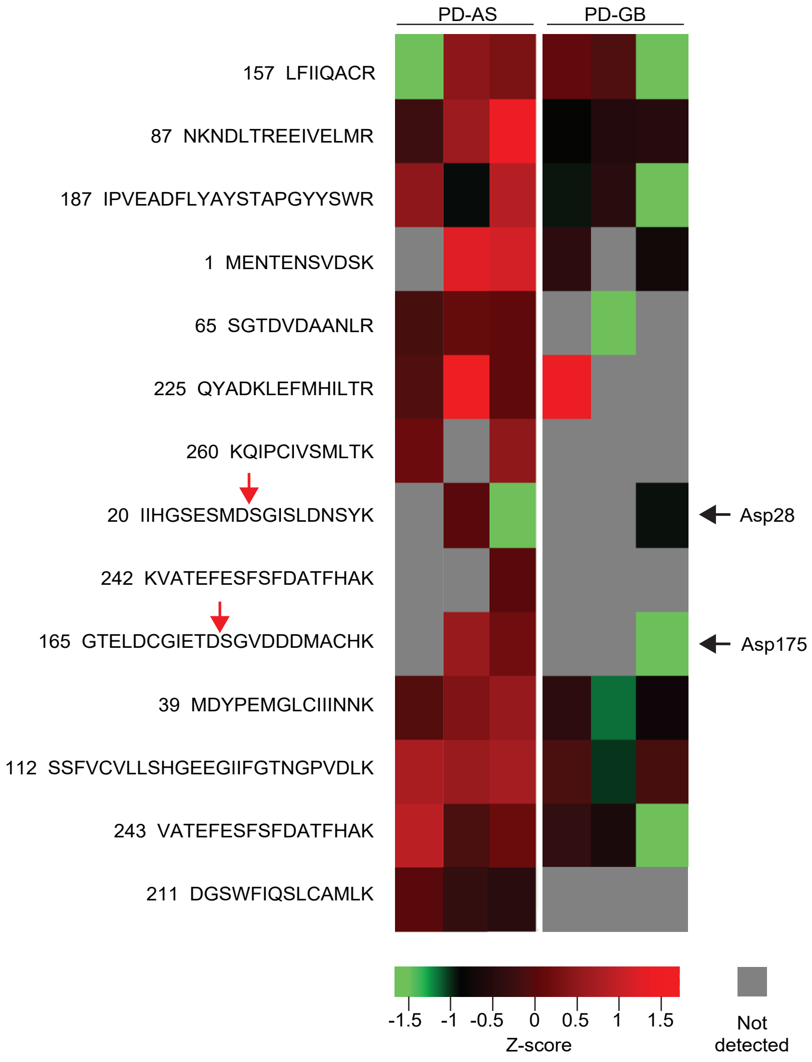

Supplementary Figure S1. Quantification of individual peptides in CASP3 in three independent replicates each of PD-AS (IDH1 mutant) and PD-GB (IDH1 wildtype). Upon apoptosis, CASP3 is cleaved at Asp28 and Asp165 (indicated by red arrows), and loss of the respective intact tryptic peptides. There was no observable loss of these tryptic peptides in PD-AS vs. PD-GB, suggesting that the near-haploid cell fraction observed in PD-AS (Figure 1 A) does not represent apoptotic cells or cell fragments.
