# Supplementary material for: Proteomic Analysis Uncovers Enhanced Inflammatory Phenotype and Distinct Metabolic Changes in IDH1 Mutant Glioma Cells
Source: Int J Mol Sci. 2025 Sep 18;26(18):9075. doi: 10.3390/ijms26189075 (PMC12470816; doi:10.3390/ijms26189075)
Supplement: Supplementary file 1 [file ijms-26-09075-s001.zip › Supplementary Table S3.pdf]

Supplementary Table S3. Common DEPs in PD-AS vs PD-GB and $U87_{MUT}$ vs $U87_{WT}$ and their potential prognostic and/or predictive value

Gene name	Protein name	Fold change*	Function and potential associations with glioma
**Up-regulated**			
PML	Isoform 5 of protein PML (PML-5)	100/100	High SOX9/STAT3/PML predicts poor glioma patient survival [1]. Organizer protein of nuclear PML bodies. The PML-5 splice variant positively regulates IFNγ signaling [2]
RB1	Retinoblastoma-associated protein	100/100	Loss of RB1 function in GBM IDH_{WT} predicts improved survival [3]
MYO1B	Unconventional myosin-Ib	100/1.7	Splice variant (MYO1B-fl) induced by SRSF1 associated with glioma tumorigenesis [4]
GNG2	Guanine nucleotide-binding protein subunit gamma-2	100/1.6	Matemathical modeling suggests that downregulation is associated with TMZ resistance [5]
PPL	Periplakin	60/1.7	Not associated with glioma. Potentially predicts poor response to pembrolizumab in melanoma [6]
RGS10	Regulator of G-protein signaling 10	18/100	Not associated with glioma. Low levels predict worse prognosis in breast cancer [7]
ASMTL	Probable bifunctional dTTP/UTP pyrophosphatase/methyltransferase protein	16.2/100	Hydrolyzes modified nucleotides and prevents their misincorporation in nucleic acids [8]
SERPINE1	Plasminogen activator inhibitor 1	7.5/1.9	High expression correlates with poor survival in high grade gliomas [9]
CD82	CD82 antigen	6.5/2.5	Upregulated in high grade gliomas. Potentially increases motility [10]
H1-1	Histone H1.1	4.2/1.6	Not specifically associated with gliomas, but chromatin is heavily dependent on DNA and histone methylation status, which is indirectly affected by IDH1 mutation
TJP1	Tight junction protein ZO-1	4.1/100	Contributes to decrease permeability of glioma-conditioned normal BBB [11]
CSTB	Cystatin-B	4.0/2.4	Thiol protease inhibitor. Higher expression in high-grade gliomas [12]
RBPMS	Isoform C of RNA-binding protein with multiple splicing	3.7/1.7	Not associated with glioma. Regulates alternative splicing of pre-mRNA involved in actin cytoskeleton and focal adhesion machineries. Positive prognostic marker in colon cancer [13]
ARFIP1	Arfaptin-1	3.4/1.6	Not associated with gliomas. Controls biogenesis of secretory granules
BCAS3	BCAS3 microtubule associated cell migration factor	3.2/100	Pro-angiogenetic. Poor prognostic factor in GBM [14]
MAN1A1	Mannosyl-oligosaccharide 1,2-alpha-mannosidase IA	2.9/1.5	Involved in the maturation of Asn-linked oligosaccharides. Inhibition of MAN1A1 by the

			antipsychotic drug penfluridol alters glycosylation of PD-L1, disrupts interactions between PD1 and PD-L1 and enhances antitumor effects of anti PD-L1 antibodies in glioma [15]
PLAA	Phospholipase A-2-activating protein	2.4/100	Not associated with glioma. Enhances cisplatin-induced apoptosis in HeLa [16] and suppresses ovarian cancer metastasis via METTL3-mediated m^6A modification of TRPC3 mRNA [17]
ALDH7A1	Alpha-aminoadipic semialdehyde dehydrogenase	2.1/1.6	Protects from oxidative stress. mRNA expression reported lower by IDH1R132H expression in U251 cells [18]. High levels of the product α-aminoadipate in glioma stem cells correlates with poor patient survival [19]
CPPED1	Serine/threonine-protein phosphatase CPPED1	2.0/17.8	Blocks cell cycle progression. Involved in glucose uptake in adipocytes. Associated with high risk and poor survival in GBM, but also with increased response to immunotherapy [20]. mRNA stability via m^6A [21]
LGALS3BP	Galectin-3-binding protein	1.9/1.9	Part of a 3-protein panel (CA9, CYFIP2, LGALS3BP) identified by LC-MS/MS and IHC, and which upregulation is associated with poor prognosis in glioma [22]
CAV1	Caveolin-1	1.8/100	Activates PI3K/Akt signaling by upregulating SERPINE1, thereby promoting proliferation and metastasis of glioblastoma [23]
DPYSL2	Dihydropyrimidinase like 2	1.8/1.6	Upregulated by HOXA11, a proposed tumor suppressor in glioma [24]
NTPCR	Cancer-related nucleoside-triphosphatase	1.7/1.5	Not associated with cancer
DST	Dystonin	1.6/100	Not associated with glioma, but upregulation mitigates cisplatin resistance in CRC cells [25]
RFTN1	Raftlin	1.5/1.6	Not associated with glioma. Lipid raft protein and inflammatory biomarker. Promotes gastric cancer progression by modulating AKT/p38 signaling [26]
**Down-regulated**			
ARMCX4	Armadillo repeat-containing X-linked protein 4	0.01/0.01	Not reported cancer-associated
KIF5A	Kinesin heavy chain isoform 5A	0.01/0.28	Downregulation by miR-503 promote glioma cell proliferation, migration, and invasion [27]
MCAM	Cell surface glycoprotein MUC18	0.01/0.41	Increases stemness and aggressiveness in glioblastoma [28]
ERAL1	GTPase ERA, mitochondrial	0.01/0.57	Not associated with glioma and weakly with cancer
LRSAM1	E3 ubiquitin-protein ligase LRSAM1	0.01/0.64	Proposed to ubiquitinate and degrade SLC40A1, thereby inducing ferroptosis in glioma stem cells [29]
TECPR1	Tectonin beta-propeller repeat-containing protein 1	0.01/0.64	Not associated with glioma and weakly with cancer

DPP7	Dipeptidyl peptidase 2	0.02/0.25	Not associated with cancer. Negative prognostic marker in CRC [30]
MSI2	RNA-binding protein Musashi homolog 2	0.02/0.52	m^6A regulator. Silencing inhibits MGMT expression, tumor growth and reversed TMZ resistance in glioblastoma [31]
ENPP4	Bis(5-adenosyl)-triphosphatase ENPP4	0.05/0.35	Not associated with glioma. Leukemia stem cell marker [32]
VCAN	Versican core protein	0.06/0.40	VCAN released from glioma promotes tumor expansion [1]
LRRC1	Leucine-rich repeat-containing protein 1	0.06/0.52	Not associated with glioma and weakly with cancer
RHOT1	Mitochondrial Rho GTPase 1	0.06/0.61	Not associated with glioma. High levels associated with short survival in gastric cancer [33]
COL7A1	Collagen alpha-1(VII) chain	0.08/0.56	Proposed to mediate increased anchoring and lower migration of glioma cells [34]
ABCC3	Canalicular multispecific organic anion transporter 2	0.11/0.38	High expression associated with poor survival and impaired response to temozolomide in glioma [35]
NES	Nestin	0.14/0.01	Glioma stem cell marker. High expression predicts shorter survival. Low expression in IDH_{MUT} gliomas [36]
HSPA4L	Heat shock 70 kDa protein 4L	0.16/0.29	Not associated with glioma and weakly with cancer
HDHD2	Haloacid dehalogenase-like hydrolase domain-containing protein 2	0.17/0.40	Not reported cancer-associated
SERPINB8	Serpin B8	0.18/0.26	Not associated with glioma and weakly with cancer
MMAB	Corrinoid adenosyltransferase MMAB	0.21/0.61	Not associated with glioma and weakly with cancer
THOC1	THO complex subunit 1	0.21/0.65	Knockout reduces GBM cell viability across patient-derived xenograft lines, apparently involving telomere shortening [37]
ASF1A	Histone chaperone ASF1A	0.23/0.60	Not associated with glioma. Promotes NHEJ in BRCA1-deficient cancer cells [38]
ALDH2	Aldehyde dehydrogenase, mitochondrial	0.24/0.38	Not associated with glioma. Low levels could lead to formaldehyde accumulation, inflammation and DNA/RNA damage [39]
SLC1A3	Excitatory amino acid transporter 1	0.28/0.20	Glioblastoma stem cells with low SLC1A3 expression are dependent on GLS to maintain appropriate cellular glutamate levels [40]
ASL	Argininosuccinate lyase	0.29/0.27	Drives activation of mutant *TERT* promoters in glioma [41]
SLC27A3	Long-chain fatty acid transport protein 3	0.31/0.26	Knockout in U87MG cells reduces malignancy and upregulates glycolysis [42] .
NINJ1	Ninjurin-1	0.32/0.45	Not associated with glioma. Stress-induced plasma membrane rupture protein. Downregulation of NINJ1 increases cystine uptake and protects against ferroptosis [43]
FDXR	NADPH:adrenodoxin oxidoreductase, mitochondrial	0.32/0.46	Strongly radiation induced. Weakly associated with glioma, but hypothesized to interact with IDH1, TP53 and ATRX [44]

IFRD2	Interferon-related developmental regulator 2	0.33/0.01	Not associated with glioma and weakly with cancer
RHOT2	Mitochondrial Rho GTPase 2	0.33/0.62	Not associated with glioma and weakly with cancer
STX16	Syntaxin-16	0.34/0.01	Not associated with glioma and weakly with cancer
CDC34	Ubiquitin-conjugating enzyme E2 R1	0.39/0.50	Not associated with glioma. Knockdown inhibits proliferation of NSCLC cells [45]
FLOT2	Flotillin-2	0.39/0.65	Silencing inhibits growth and invasion of gliomas cells [46]
CNN3	Calponin-3	0.41/0.54	Lower expression in IDH_{MUT} than IDH_{WT} gliomas. High expression associated with reduced survival [47]
POLR2I	DNA-directed RNA polymerase II subunit RPB9	0.41/0.58	Not associated with glioma and weakly with other cancers
SNX27	Sorting nexin-27	0.44/0.39	Not associated with glioma and weakly with other cancers
SMARCD1	SWI/SNF-related matrix-associated actin-dependent regulator of chromatin subfamily D member 1	0.44/0.41	Depletion promotes proliferation, invasion, and chemoresistance in glioma cells [48]
ESRRA	Steroid hormone receptor ERR1	0.46/0.52	IRE1 inhibition and hypoxia strongly downregulates ESRRA in glioma cells [1]. Pharmacological inhibition of ESRRA sensitizes esophageal adenocarcinoma organoids and patient-derived xenografts to radiation [49]
CDC42SE1	CDC42 small effector protein 1	0.47/0.29	Not associated with glioma. Upregulation reduces proliferation of squamous carcinoma cells [50]
POGZ	Pogo transposable element with ZNF domain	0.47/0.34	Not associated with glioma. Suppresses metastasis in triple negative breast cancer by attenuating TGFβ signaling [51]
DDR2	Discoidin domain-containing receptor 2	0.47/0.37	Case report of likely inactivating mutation of DDR2 (Leu338Phe) in glioblastoma, with exceptional response to first-line TMZ [52]
IQGAP3	Ras GTPase-activating-like protein IQGAP3	0.47/0.55	High expression in gliomas is associated with an unfavorable prognosis. Mediates immunosuppressive microenvironment in glioblastoma [53]
GNG5	Guanine nucleotide-binding protein G(I)/G(S)/G(O) subunit gamma-5	0.48/0.45	Oncogene in glioma, promoting migration, proliferation stemness and glycolysis [54,55]
IAH1	Isoamyl acetate-hydrolyzing esterase 1 homolog	0.49/0.50	Not reported cancer-associated
NNT	NAD(P) transhydrogenase, mitochondrial	0.50/0.48	Not associated with glioma. In gastric cancer, acetylated NNT boosts NADPH production and protects against ferroptosis [56]
ATXN10	Ataxin-10	0.50/0.61	Not associated with glioma and weakly with cancer
PRR12	Proline-rich protein 12	0.52/0.01	Overexpression induces apoptosis in glioma cells [57]
TUBB3	Tubulin beta-3 chain	0.54/0.64	Neuronal and stem cell marker. Weakly associated with glioma.
FECH	Ferrochelatase, mitochondrial	0.55/0.35	Low levels enhance 5-ALA-based fluorescence and photodynamic therapy efficacy in glioma [58]

NEDD4L	E3 ubiquitin-protein ligase NEDD4-like	0.56/0.37	Low levels in glioma correlates with poor prognosis [59,60]
TFB2M	Dimethyladenosine transferase 2, mitochondrial	0.56/0.56	Not associated with glioma. High expression facilitates cell growth and metastasis in HCC [61]
CXADR	Coxsackievirus and adenovirus receptor	0.57/0.04	Hypoxia-induced in glioma. Reduces migration via stabilization of microtubules [62]
DHX8	ATP-dependent RNA helicase DHX8	0.57/0.62	Not associated with glioma and weakly with cancer
ME2	NAD-dependent malic enzyme, mitochondrial	0.58/0.49	Promotes proneural-mesenchymal transition and lipogenesis in glioblastoma [63]
PLOD3	Procollagen-lysine,2-oxoglutarate 5-dioxygenase 3	0.58/0.52	m^7G-related gene. 2OG-dependent and thus inhibited in IDH_{MUT}. High levels accelerate tumor progression and indicate poor prognosis [64]
SNRPD2	Small nuclear ribonucleoprotein Sm D2	0.60/0.62	Not associated with glioma. Can disrupt liquid-liquid phase separation during polyA-lengthening by interfering with PABPN1 [65]
TXNL1	Thioredoxin-like protein 1	0.61/0.41	Not associated with glioma. Downregulation mediates cisplatin resistance in gastric cancer cells [66]
SUMF2	Inactive C-alpha-formylglycine-generating enzyme 2	0.61/0.48	Not associated with glioma and weakly with cancer
PCK2	Phosphoenolpyruvate carboxykinase [GTP], mitochondrial	0.64/0.40	High expression linked to shorter survival, especially in LGGs [67]
CRLF3	Isoform 2 of Cytokine receptor-like factor 3	0.65/0.47	Not associated with glioma and weakly with cancer

*Linear fold change PD-AS vs PD-GB/$U87_{MUT}$ vs $U87_{WT}$

References

1. Aldaz, P.; Martin-Martin, N.; Saenz-Antonanzas, A.; Carrasco-Garcia, E.; Alvarez-Satta, M.; Elua-Pinin, A.; Pollard, S.M.; Lawrie, C.H.; Moreno-Valladares, M.; Sampron, N.; et al. High SOX9 Maintains Glioma Stem Cell Activity through a Regulatory Loop Involving STAT3 and PML. *Int J Mol Sci* **2022**, *23*, doi:10.3390/ijms23094511.
2. Nisole, S.; Maroui, M.A.; Mascle, X.H.; Aubry, M.; Chelbi-Alix, M.K. Differential Roles of PML Isoforms. *Front Oncol* **2013**, *3*, 125, doi:10.3389/fonc.2013.00125.
3. Dono, A.; Ramesh, A.V.; Wang, E.; Shah, M.; Tandon, N.; Ballester, L.Y.; Esquenazi, Y. The role of RB1 alteration and 4q12 amplification in IDH-WT glioblastoma. *Neurooncol Adv* **2021**, *3*, vdab050, doi:10.1093/noajnl/vdab050.
4. Zhou, X.; Wang, R.; Li, X.; Yu, L.; Hua, D.; Sun, C.; Shi, C.; Luo, W.; Rao, C.; Jiang, Z.; et al. Splicing factor SRSF1 promotes gliomagenesis via oncogenic splice-switching of MYO1B. *J Clin Invest* **2019**, *129*, 676–693, doi:10.1172/JCI120279.
5. Nayak, R.; Mallick, B. LncRNA-associated competing endogenous RNA network analysis uncovered key lncRNAs involved in temozolomide resistance and tumor recurrence of glioblastoma. *J Mol Recognit* **2023**, *36*, e3060, doi:10.1002/jmr.3060.
6. Edmonds, N.L.; Flores, S.E.; Mahmutovic, A.; Young, S.J.; Mauldin, I.S.; Slingluff, C.L., Jr. CD103 and periplakin are potential biomarkers for response of metastatic melanoma to pembrolizumab. *Melanoma Res* **2022**, *32*, 440–450, doi:10.1097/CMR.0000000000000855.

7. Liu, Y.; Jiang, Y.; Qiu, P.; Ma, T.; Bai, Y.; Bu, J.; Hu, Y.; Jin, M.; Zhu, T.; Gu, X. RGS10 deficiency facilitates distant metastasis by inducing epithelial-mesenchymal transition in breast cancer. *Elife* **2024**, *13*, doi:10.7554/eLife.97327.
8. Tchigvintsev, A.; Tchigvintsev, D.; Flick, R.; Popovic, A.; Dong, A.; Xu, X.; Brown, G.; Lu, W.; Wu, H.; Cui, H.; et al. Biochemical and structural studies of conserved Maf proteins revealed nucleotide pyrophosphatases with a preference for modified nucleotides. *Chem Biol* **2013**, *20*, 1386–1398, doi:10.1016/j.chembiol.2013.09.011.
9. Connolly, N.P.; Galisteo, R.; Xu, S.; Bar, E.E.; Peng, S.; Tran, N.L.; Ames, H.M.; Kim, A.J.; Woodworth, G.F.; Winkles, J.A. Elevated fibroblast growth factor-inducible 14 expression transforms proneural-like gliomas into more aggressive and lethal brain cancer. *Glia* **2021**, *69*, 2199–2214, doi:10.1002/glia.24018.
10. Paradowski, M.; Bilinska, M.; Bar, J. Characteristics of the expression of KAI1/CD82 and PDGFRbeta and their impact on glioma progression. *Folia Neuropathol* **2016**, *54*, 241–248, doi:10.5114/fn.2016.62554.
11. Leng, X.; Ma, J.; Liu, Y.; Shen, S.; Yu, H.; Zheng, J.; Liu, X.; Liu, L.; Chen, J.; Zhao, L.; et al. Mechanism of piR-DQ590027/MIR17HG regulating the permeability of glioma conditioned normal BBB. *J Exp Clin Cancer Res* **2018**, *37*, 246, doi:10.1186/s13046-018-0886-0.
12. Zhang, R.; Tremblay, T.L.; McDermid, A.; Thibault, P.; Stanimirovic, D. Identification of differentially expressed proteins in human glioblastoma cell lines and tumors. *Glia* **2003**, *42*, 194–208, doi:10.1002/glia.10222.
13. Gyorffy, B. Integrated analysis of public datasets for the discovery and validation of survival-associated genes in solid tumors. *Innovation (Camb)* **2024**, *5*, 100625, doi:10.1016/j.xinn.2024.100625.
14. Wang, Y.; Li, Y.; Sun, Q.; Yuan, F.; Xu, Y.; Tong, S.; Li, Y.; Yi, S.; Yan, T.; Chen, Q.; et al. BCAS3 accelerates glioblastoma tumorigenesis by restraining the P53/GADD45alpha signaling pathway. *Exp Cell Res* **2022**, *417*, 113231, doi:10.1016/j.yexcr.2022.113231.
15. Xu, W.; Wang, Y.; Zhang, N.; Lin, X.; Zhu, D.; Shen, C.; Wang, X.; Li, H.; Xue, J.; Yu, Q.; et al. The Antipsychotic Drug Penfluridol Inhibits N-Linked Glycoprotein Processing and Enhances T-cell-Mediated Tumor Immunity. *Mol Cancer Ther* **2024**, *23*, 648–661, doi:10.1158/1535-7163.MCT-23-0449.
16. Zhang, F.; Suarez, G.; Sha, J.; Sierra, J.C.; Peterson, J.W.; Chopra, A.K. Phospholipase A2-activating protein (PLAA) enhances cisplatin-induced apoptosis in HeLa cells. *Cell Signal* **2009**, *21*, 1085–1099, doi:10.1016/j.cellsig.2009.02.018.
17. Shen, Z.; Gu, L.; Liu, Y.; Wang, L.; Zhu, J.; Tang, S.; Wei, X.; Wang, J.; Zhang, S.; Wang, X.; et al. PLAA suppresses ovarian cancer metastasis via METTL3-mediated m(6)A modification of TRPC3 mRNA. *Oncogene* **2022**, *41*, 4145–4158, doi:10.1038/s41388-022-02411-w.
18. Sauers, M.E.; Abraham, R.T.; Alvin, J.D.; Zemaitis, M.A. Factors influencing dimethylphenobarbital N-demethylation by isolated hepatocytes from untreated and phenobarbital-treated rats. *Drug Metab Dispos* **1980**, *8*, 208–211.
19. Rosi, A.; Ricci-Vitiani, L.; Biffoni, M.; Grande, S.; Luciani, A.M.; Palma, A.; Runci, D.; Cappellari, M.; De Maria, R.; Guidoni, L.; et al. (1) H NMR spectroscopy of glioblastoma stem-like cells identifies alpha-aminoadipate as a marker of tumor aggressiveness. *NMR Biomed* **2015**, *28*, 317–326, doi:10.1002/nbm.3254.
20. Sun, G.; Liu, W. The neutrophil extracellular traps-related gene signature predicts the prognosis of glioblastoma multiforme. *Folia Neuropathol* **2024**, *62*, 59–75, doi:10.5114/fn.2023.132980.
21. Li, J.; Cao, H.; Yang, J.; Wang, B. CircCDK1 blocking IGF2BP2-mediated m6A modification of CPPED1 promotes laryngeal squamous cell carcinoma metastasis via the PI3K/AKT signal pathway. *Gene* **2023**, *884*, 147686, doi:10.1016/j.gene.2023.147686.

22. Hudson, A.L.; Cho, A.; Colvin, E.K.; Hayes, S.A.; Wheeler, H.R.; Howell, V.M. CA9, CYFIP2 and LGALS3BP-A Novel Biomarker Panel to Aid Prognostication in Glioma. *Cancers (Basel)* **2024**, *16*, doi:10.3390/cancers16051069.
23. Wang, Z.; Chen, G.; Yuan, D.; Wu, P.; Guo, J.; Lu, Y.; Wang, Z. Caveolin-1 promotes glioma proliferation and metastasis by enhancing EMT via mediating PAI-1 activation and its correlation with immune infiltrates. *Heliyon* **2024**, *10*, e24464, doi:10.1016/j.heliyon.2024.e24464.
24. Se, Y.B.; Kim, S.H.; Kim, J.Y.; Kim, J.E.; Dho, Y.S.; Kim, J.W.; Kim, Y.H.; Woo, H.G.; Kim, S.H.; Kang, S.H.; et al. Underexpression of HOXA11 Is Associated with Treatment Resistance and Poor Prognosis in Glioblastoma. *Cancer Res Treat* **2017**, *49*, 387–398, doi:10.4143/crt.2016.106.
25. Yu, J.; Deng, X.; Lin, X.; Xie, L.; Guo, S.; Lin, X.; Lin, D. DST regulates cisplatin resistance in colorectal cancer via PI3K/Akt pathway. *J Pharm Pharmacol* **2024**, doi:10.1093/jpp/rgae104.
26. Deng, C.; Zhang, L.; Ma, X.; Cai, S.; Jia, Y.; Zhao, L. RFTN1 facilitates gastric cancer progression by modulating AKT/p38 signaling pathways. *Pathol Res Pract* **2022**, *234*, 153902, doi:10.1016/j.prp.2022.153902.
27. Wang, X.S.; Yu, X.J.; Wei, K.; Wang, S.X.; Liu, Q.K.; Wang, Y.G.; Li, H.; Huang, C. Mesenchymal stem cells shuttling miR-503 via extracellular vesicles enhance glioma immune escape. *Oncoimmunology* **2022**, *11*, 1965317, doi:10.1080/2162402X.2021.1965317.
28. Liang, Y.; Voshart, D.; Paridaen, J.; Oosterhof, N.; Liang, D.; Thiruvalluvan, A.; Zuhorn, I.S.; den Dunnen, W.F.A.; Zhang, G.; Lin, H.; et al. CD146 increases stemness and aggressiveness in glioblastoma and activates YAP signaling. *Cell Mol Life Sci* **2022**, *79*, 398, doi:10.1007/s00018-022-04420-0.
29. Mansuer, M.; Zhou, L.; Wang, C.; Gao, L.; Jiang, Y. Erianin induces ferroptosis in GSCs via REST/LRSAM1 mediated SLC40A1 ubiquitination to overcome TMZ resistance. *Cell Death Dis* **2024**, *15*, 522, doi:10.1038/s41419-024-06902-4.
30. Shang, Z.; Lai, Y.; Cheng, H. DPP2/7 is a Potential Predictor of Prognosis and Target in Immunotherapy in Colorectal Cancer: An Integrative Multi-omics Analysis. *Comb Chem High Throughput Screen* **2024**, *27*, 1642–1660, doi:10.2174/0113862073290831240229060932.
31. Jiang, X.; Tan, J.; Wen, Y.; Liu, W.; Wu, S.; Wang, L.; Wangou, S.; Liu, D.; Du, C.; Zhu, B.; et al. MSI2-TGF-beta/TGF-beta R1/SMAD3 positive feedback regulation in glioblastoma. *Cancer Chemother Pharmacol* **2019**, *84*, 415–425, doi:10.1007/s00280-019-03892-5.
32. Mohd Amin, A.; Panneerselvan, N.; Md Noor, S.; Mohtaruddin, N.; Sathar, J.; Norbaya, W.S.; Osman, R.; Kee, L.H.; Mohd Yaakub, W.H.; Cheong, S.K.; et al. ENPP4 and HOXA3 as potential leukaemia stem cell markers in acute myeloid leukaemia. *Malays J Pathol* **2023**, *45*, 65–76.
33. Kong, F.; Yang, S.; Shi, R.; Peng, Y. The Up-Regulated Expression of Mitochondrial Membrane Molecule RHOT1 in Gastric Cancer Predicts the Prognosis of Patients and Promotes the Malignant Biological Behavior of Cells. *Mol Biotechnol* **2024**, doi:10.1007/s12033-024-01107-8.
34. Motaln, H.; Gruden, K.; Hren, M.; Schichor, C.; Primon, M.; Rotter, A.; Lah, T.T. Human mesenchymal stem cells exploit the immune response mediating chemokines to impact the phenotype of glioblastoma. *Cell Transplant* **2012**, *21*, 1529–1545, doi:10.3727/096368912X640547.
35. Ruiz-Lopez, E.; Jovcevska, I.; Gonzalez-Gomez, R.; Tejero, H.; Al-Shahrour, F.; Muyldermans, S.; Schuhmacher, A.J. Nanobodies targeting ABCC3 for immunotargeted applications in glioblastoma. *Sci Rep* **2022**, *12*, 22581, doi:10.1038/s41598-022-27161-3.
36. An, S.; Song, I.H.; Woo, C.G. Diagnostic Value of Nestin Expression in Adult Gliomas. *Int J Surg Pathol* **2023**, *31*, 1014–1020, doi:10.1177/10668969221125792.
37. Budhiraja, S.; Baisiwala, S.; Cho, S.; Chojak, R.; Kazi, H.A.; Stepniak, A.; Perrault, E.N.; Chen, L.; Park, C.H.; Dmello, C.; et al. THOC1 complexes with SIN3A to regulate R-loops and promote glioblastoma progression. *bioRxiv* **2024**, doi:10.1101/2024.09.24.614748.

38. Tang, M.; Chen, Z.; Wang, C.; Feng, X.; Lee, N.; Huang, M.; Zhang, H.; Li, S.; Xiong, Y.; Chen, J. Histone chaperone ASF1 acts with RIF1 to promote DNA end joining in BRCA1-deficient cells. *J Biol Chem* **2022**, *298*, 101979, doi:10.1016/j.jbc.2022.101979.
39. Rieckher, M.; Gallrein, C.; Alquezar-Artieda, N.; Bourached-Silva, N.; Vaddavalli, P.L.; Mares, D.; Backhaus, M.; Blindauer, T.; Greger, K.; Wiesner, E.; et al. Distinct DNA repair mechanisms prevent formaldehyde toxicity during development, reproduction and aging. *Nucleic Acids Res* **2024**, *52*, 8271–8285, doi:10.1093/nar/gkae519.
40. Restall, I.J.; Cseh, O.; Richards, L.M.; Pugh, T.J.; Luchman, H.A.; Weiss, S. Brain Tumor Stem Cell Dependence on Glutaminase Reveals a Metabolic Vulnerability through the Amino Acid Deprivation Response Pathway. *Cancer Res* **2020**, *80*, 5478–5490, doi:10.1158/0008-5472.CAN-19-3923.
41. Shi, Z.; Ge, X.; Li, M.; Yin, J.; Wang, X.; Zhang, J.; Chen, D.; Li, X.; Wang, X.; Ji, J.; et al. Argininosuccinate lyase drives activation of mutant TERT promoter in glioblastomas. *Mol Cell* **2022**, *82*, 3919–3931 e3917, doi:10.1016/j.molcel.2022.09.024.
42. Kolar, E.A.; Shi, X.; Clay, E.M.; Liu, Y.; Xia, S.; Zhang, C.; Le, A.; Watkins, P.A. Depleting glioblastoma cells of very long-chain acyl-CoA synthetase 3 (ACSVL3) produces metabolic alterations in non-lipid pathways. *bioRxiv* **2023**, doi:10.1101/2023.09.18.558236.
43. Chen, S.Y.; Wu, J.; Chen, Y.; Wang, Y.E.; Setayeshpour, Y.; Federico, C.; Mestre, A.A.; Lin, C.C.; Chi, J.T. NINJ1 regulates ferroptosis via xCT antiporter interaction and CoA modulation. *Cell Death Dis* **2024**, *15*, 755, doi:10.1038/s41419-024-07135-1.
44. Xie, Y.; Tan, Y.; Yang, C.; Zhang, X.; Xu, C.; Qiao, X.; Xu, J.; Tian, S.; Fang, C.; Kang, C. Omics-based integrated analysis identified ATRX as a biomarker associated with glioma diagnosis and prognosis. *Cancer Biol Med* **2019**, *16*, 784–796, doi:10.20892/j.issn.2095-3941.2019.0143.
45. Zhao, X.C.; Wang, G.Z.; Wen, Z.S.; Zhou, Y.C.; Hu, Q.; Zhang, B.; Qu, L.W.; Gao, S.H.; Liu, J.; Ma, L.; et al. Systematic identification of CDC34 that functions to stabilize EGFR and promote lung carcinogenesis. *EBioMedicine* **2020**, *53*, 102689, doi:10.1016/j.ebiom.2020.102689.
46. Song, T.; Hu, Z.; Liu, J.; Huang, W. FLOT2 upregulation promotes growth and invasion by interacting and stabilizing EphA2 in gliomas. *Biochem Biophys Res Commun* **2021**, *548*, 67–73, doi:10.1016/j.bbrc.2021.02.062.
47. Xie, Y.; Ding, W.; Xiang, Y.; Wang, X.; Yang, J. Calponin 3 Acts as a Potential Diagnostic and Prognostic Marker and Promotes Glioma Cell Proliferation, Migration, and Invasion. *World Neurosurg* **2022**, *165*, e721–e731, doi:10.1016/j.wneu.2022.06.136.
48. Zhu, Y.; Wang, H.; Fei, M.; Tang, T.; Niu, W.; Zhang, L. Smarcd1 Inhibits the Malignant Phenotypes of Human Glioblastoma Cells via Crosstalk with Notch1. *Mol Neurobiol* **2021**, *58*, 1438–1452, doi:10.1007/s12035-020-02190-z.
49. Dings, M.P.G.; van der Zalm, A.P.; Bootsma, S.; van Maanen, T.F.J.; Waasdorp, C.; van den Ende, T.; Liu, D.; Bailey, P.; Koster, J.; Zwijnenburg, D.A.; et al. Estrogen-related receptor alpha drives mitochondrial biogenesis and resistance to neoadjuvant chemoradiation in esophageal cancer. *Cell Rep Med* **2022**, *3*, 100802, doi:10.1016/j.xcrm.2022.100802.
50. Kalailingam, P.; Tan, H.B.; Pan, J.Y.; Tan, S.H.; Thanabalu, T. Overexpression of CDC42SE1 in A431 Cells Reduced Cell Proliferation by Inhibiting the Akt Pathway. *Cells* **2019**, *8*, doi:10.3390/cells8020117.
51. Heath, J.; Mirabelli, C.; Annis, M.G.; Sabourin, V.; Hebert, S.; Findlay, S.; Kim, H.; Witcher, M.; Kleinman, C.L.; Siegel, P.M.; et al. The Neurodevelopmental Protein POGZ Suppresses Metastasis in Triple Negative Breast Cancer by Attenuating TGFbeta Signaling. *Cancer Res* **2024**, doi:10.1158/0008-5472.CAN-23-3887.
52. El Husseini, K.; Marguet, F.; Lamy, A.; Magne, N.; Fontanilles, M. Major response to temozolomide as first-line treatment for newly-diagnosed DDR2-mutated glioblastoma: A case report. *Rev Neurol (Paris)* **2020**, *176*, 402–404, doi:10.1016/j.neurol.2019.09.007.

53. Gao, X.; Ge, J.; Gao, X.; Mei, N.A.; Su, Y.; Shan, S.; Qian, W.; Guan, J.; Zhang, Z.; Wang, L. IQGAP3 promotes the progression of glioma as an immune and prognostic marker. *Oncol Res* **2024**, *32*, 659–678, doi:10.32604/or.2023.046712.
54. Zhang, W.; Liu, Z.; Liu, B.; Jiang, M.; Yan, S.; Han, X.; Shen, H.; Na, M.; Wang, Y.; Ren, Z.; et al. GNG5 is a novel oncogene associated with cell migration, proliferation, and poor prognosis in glioma. *Cancer Cell Int* **2021**, *21*, 297, doi:10.1186/s12935-021-01935-7.
55. Liang, S.; Zhu, L.; Yang, F.; Dong, H. Transcription factor YY1-activated GNG5 facilitates glioblastoma cell growth, invasion, stemness and glycolysis through Wnt/beta-catenin pathway. *Sci Rep* **2024**, *14*, 25234, doi:10.1038/s41598-024-76019-3.
56. Han, Y.; Zhang, Y.Y.; Pan, Y.Q.; Zheng, X.J.; Liao, K.; Mo, H.Y.; Sheng, H.; Wu, Q.N.; Liu, Z.X.; Zeng, Z.L.; et al. IL-1beta-associated NNT acetylation orchestrates iron-sulfur cluster maintenance and cancer immunotherapy resistance. *Mol Cell* **2023**, *83*, 1887–1902 e1888, doi:10.1016/j.molcel.2023.05.011.
57. Mao, Y.; Shen, G.; Su, Z.; Du, J.; Xu, F.; Yu, Y. RAD21 inhibited transcription of tumor suppressor MIR4697HG and led to glioma tumorigenesis. *Biomed Pharmacother* **2020**, *123*, 109759, doi:10.1016/j.biopha.2019.109759.
58. Teng, L.; Nakada, M.; Zhao, S.G.; Endo, Y.; Furuyama, N.; Nambu, E.; Pyko, I.V.; Hayashi, Y.; Hamada, J.I. Silencing of ferrochelatase enhances 5-aminolevulinic acid-based fluorescence and photodynamic therapy efficacy. *Br J Cancer* **2011**, *104*, 798–807, doi:10.1038/bjc.2011.12.
59. Nie, X.H.; Qiu, S.; Xing, Y.; Xu, J.; Lu, B.; Zhao, S.F.; Li, Y.T.; Su, Z.Z. Paeoniflorin Regulates NEDD4L/STAT3 Pathway to Induce Ferroptosis in Human Glioma Cells. *J Oncol* **2022**, *2022*, 6093216, doi:10.1155/2022/6093216.
60. Wang, H.; Zhao, B.; Bian, E.; Zong, G.; He, J.; Wang, Y.; Ma, C.; Wan, J. Ubiquitination Destabilizes Protein Sphingosine Kinase 2 to Regulate Glioma Malignancy. *Front Cell Neurosci* **2021**, *15*, 660354, doi:10.3389/fncel.2021.660354.
61. Geng, X.; Geng, Z.; Li, H.; Zhang, Y.; Li, J.; Chang, H. Over-expression of TFB2M facilitates cell growth and metastasis via activating ROS-Akt-NF-kappaB signalling in hepatocellular carcinoma. *Liver Int* **2020**, *40*, 1756–1769, doi:10.1111/liv.14440.
62. Fok, P.T.; Huang, K.C.; Holland, P.C.; Nalbantoglu, J. The Coxsackie and adenovirus receptor binds microtubules and plays a role in cell migration. *J Biol Chem* **2007**, *282*, 7512–7521, doi:10.1074/jbc.M607230200.
63. Yang, M.; Chen, X.; Zhang, J.; Xiong, E.; Wang, Q.; Fang, W.; Li, L.; Fei, F.; Gong, A. ME2 Promotes Proneural-Mesenchymal Transition and Lipogenesis in Glioblastoma. *Front Oncol* **2021**, *11*, 715593, doi:10.3389/fonc.2021.715593.
64. Tsai, C.K.; Huang, L.C.; Tsai, W.C.; Huang, S.M.; Lee, J.T.; Hueng, D.Y. Overexpression of PLOD3 promotes tumor progression and poor prognosis in gliomas. *Oncotarget* **2018**, *9*, 15705–15720, doi:10.18632/oncotarget.24594.
65. Hu, Z.; Li, M.; Chen, Y.; Chen, L.; Han, Y.; Chen, C.; Lu, X.; You, N.; Lou, Y.; Huang, Y.; et al. Disruption of PABPN1 phase separation by SNRPD2 drives colorectal cancer cell proliferation and migration through promoting alternative polyadenylation of CTNNBIP1. *Sci China Life Sci* **2024**, *67*, 1212–1225, doi:10.1007/s11427-023-2495-x.
66. Ni, P.; Xu, W.; Zhang, Y.; Chen, Q.; Li, A.; Wang, S.; Xu, S.; Zhou, J. TXNL1 induces apoptosis in cisplatin resistant human gastric cancer cell lines. *Curr Cancer Drug Targets* **2015**, *14*, 850–859, doi:10.2174/1568009614666141028094612.
67. Xue, S.; Cai, Y.; Liu, J.; Ji, K.; Yi, P.; Long, H.; Zhang, X.; Li, P.; Song, Y. Dysregulation of phosphoenolpyruvate carboxykinase in cancers: A comprehensive analysis. *Cell Signal* **2024**, *120*, 111198, doi:10.1016/j.cellsig.2024.111198.
