# Supplementary material for: Proteomic Analysis Uncovers Enhanced Inflammatory Phenotype and Distinct Metabolic Changes in IDH1 Mutant Glioma Cells
Source: Int J Mol Sci. 2025 Sep 18;26(18):9075. doi: 10.3390/ijms26189075 (PMC12470816; doi:10.3390/ijms26189075)
Supplement: Supplementary file 1 [file ijms-26-09075-s001.zip › Supplementary Table S4.pdf]

Supplementary Table S4. 2OG-dependent histone demethylases, their targets and reported functional consequences of their inhibition

**Gene name**	**Putative primary cancer role**	**Primary targets**	**Functional consequences if demethylase is inhibited or downregulated**	**PD-AS vs PD-GB***	**$U87_{MUT}$ vs $U87_{WT}$**
KDM2A	Pro- and antioncogenic	H3K36me1/2	H3K36me2 enhances recruitment of NHEJ factors (NBS1 and Ku70) to DSBs [1].	NS (14.3)	NS (14.9)
KDM2B	Pro- and antioncogenic	H3K4me3 H3K36me2	Inhibition of glioma cell proliferation and G0/G1 arrest [1]	ND	ND
KDM3A	Oncogene	H3K9me1 H3K9me2	May enhance resistance to TMZ in glioma [2]	ND	ND
KDM3B	Tumor suppressor	H3K9me1 H3K9me2 H4R3me2s	Different consequences across cancers [3]	NS (18.3)	NS (15.3)
KDM4A	Oncogene	H3K9me3 H3K36me3	Activation of genes with ASCL2 and E2F-responsive promoters. Silencing downregulates MYC [4]	0.24 (14.1)	ND
KDM4B	Oncogene	H3K9me3 H3K36me3	Silencing downregulates MYC in GB [4]	NS (16.9)	ND
KDM4C	Oncogene	H3K9me3 H3K36me3	Loss of viability of glioblastoma initiating cells (GICs) [5]	ND	ND
KDM5A	Pro- and antioncogenic	H3K4me2 H3K4me3	May both stimulate and repress promotors. Acts via NURF chromatin remodeling	NS (15.0)	ND
KDM5B	Oncogene	H3K4me1 H3K4me2 H3K4me3	Transcriptional activation	NS (15.6)	ND
KDM5C	Oncogene	H3K4me2 H3K4me3		NS (15.8)	ND
KDM6A	Tumor suppressor	H3K27me2 H3K27me3	Activation of inflammatory responses, incl. T-box genes. TBX21 associated with increased inflammation and expr of HLA genes and high risk, but can also signify response to immune therapy [6]	ND	ND
KDM6B	Pro- and antioncogenic	H3K27me2 H3K27me3	As KDM6A, but KBM6B especially vulnerable to low 2OG [7]	ND	ND
KDM7A	Oncogene	H3K9me2 H3K27me2	Loss of viability of glioblastoma initiating cells (GICs) [5]	ND	ND
PHF2	Tumor suppressor	H3K9me2	Mixed effects in different cancers [8]	NS (15.7)	ND
PHF8	Oncogene	H3K9me2 H3K27me2 (H3K9me1)	PHF8 has tumor suppressing activities in several cancers [8]	NS (12.2)	NS (14.1)
RIOX1	Tumor suppressor	H3K4me3 H3K36me3	RIOX1 depletion enhances homologous recombination (HR) repair but not nonhomologous end-joining (NHEJ) repair in irradiated [9]	ND	100 (15.8)

* NS: Non-significant difference (log_2 MS intensities of IDH mutants are given in parentheses, and higher intensities are colored darker red); ND: Not detected

References

1. Fnu, S.; Williamson, E.A.; De Haro, L.P.; Brenneman, M.; Wray, J.; Shaheen, M.; Radhakrishnan, K.; Lee, S.H.; Nickoloff, J.A.; Hromas, R. Methylation of histone H3 lysine 36 enhances DNA repair by nonhomologous end-joining. *Proc Natl Acad Sci U S A* **2011**, *108*, 540–545, doi:10.1073/pnas.1013571108.

2. Li, T.; Fu, X.; Wang, J.; Shang, W.; Wang, X.; Zhang, L.; Li, J. Mechanism of NURP1 in temozolomide resistance in hypoxia-treated glioma cells via the KDM3A/TFEB axis. *Oncol Res* **2023**, *31*, 345–359, doi:10.32604/or.2023.028724.
3. Yoo, J.; Kim, G.W.; Jeon, Y.H.; Lee, S.W.; Kwon, S.H. Epigenetic roles of KDM3B and KDM3C in tumorigenesis and their therapeutic implications. *Cell Death Dis* **2024**, *15*, 451, doi:10.1038/s41419-024-06850-z.
4. Wang, Z.; Cai, H.; Li, Z.; Sun, W.; Zhao, E.; Cui, H. Histone demethylase KDM4B accelerates the progression of glioblastoma via the epigenetic regulation of MYC stability. *Clin Epigenetics* **2023**, *15*, 192, doi:10.1186/s13148-023-01608-4.
5. Mallm, J.P.; Windisch, P.; Biran, A.; Gal, Z.; Schumacher, S.; Glass, R.; Herold-Mende, C.; Meshorer, E.; Barbus, M.; Rippe, K. Glioblastoma initiating cells are sensitive to histone demethylase inhibition due to epigenetic deregulation. *Int J Cancer* **2020**, *146*, 1281–1292, doi:10.1002/ijc.32649.
6. Long, C.; Song, Y.; Pan, Y.; Wu, C. Identification of molecular subtypes and a risk model based on inflammation-related genes in patients with low grade glioma. *Heliyon* **2023**, *9*, e22429, doi:10.1016/j.heliyon.2023.e22429.
7. Laukka, T.; Myllykoski, M.; Looper, R.E.; Koivunen, P. Cancer-associated 2-oxoglutarate analogues modify histone methylation by inhibiting histone lysine demethylases. *J Mol Biol* **2018**, *430*, 3081–3092, doi:10.1016/j.jmb.2018.06.048.
8. Shao, P.; Liu, Q.; Qi, H.H. KDM7 Demethylases: Regulation, Function and Therapeutic Targeting. *Adv Exp Med Biol* **2023**, *1433*, 167–184, doi:10.1007/978-3-031-38176-8_8.
9. Xiao, Y.; Li, J.; Liao, X.; He, Y.; He, T.; Yang, C.; Jiang, L.; Jeon, S.M.; Lee, J.H.; Chen, Y.; et al. RIOX1-demethylated cGAS regulates ionizing radiation-elicited DNA repair. *Bone Res* **2022**, *10*, 19, doi:10.1038/s41413-022-00194-0.
